# Rapid Alterations in Perirenal Adipose Tissue Transcriptomic Networks with Cessation of Voluntary Running

**DOI:** 10.1371/journal.pone.0145229

**Published:** 2015-12-17

**Authors:** Gregory N. Ruegsegger, Joseph M. Company, Ryan G. Toedebusch, Christian K. Roberts, Michael D. Roberts, Frank W. Booth

**Affiliations:** 1 Department of Biomedical Science, College of Veterinary Medicine, University of Missouri, Columbia, Missouri, United States of America; 2 Geriatrics, Research, Education and Clinical Center, VA of Greater Los Angeles Healthcare System, Los Angeles, California, United States of America; 3 Department of Medical Pharmacology and Physiology, University of Missouri, Columbia, Missouri, United States of America; 4 Department of Nutrition and Exercise Physiology, University of Missouri, Columbia, Missouri, United States of America; 5 Dalton Cardiovascular Research Center, University of Missouri, Columbia, Missouri, United States of America; University of California, San Francisco, UNITED STATES

## Abstract

In maturing rats, the growth of abdominal fat is attenuated by voluntary wheel running. After the cessation of running by wheel locking, a rapid increase in adipose tissue growth to a size that is similar to rats that have never run (i.e. catch-up growth) has been previously reported by our lab. In contrast, diet-induced increases in adiposity have a slower onset with relatively delayed transcriptomic responses. The purpose of the present study was to identify molecular pathways associated with the rapid increase in adipose tissue after ending 6 wks of voluntary running at the time of puberty. Age-matched, male Wistar rats were given access to running wheels from 4 to 10 weeks of age. From the 10^th^ to 11^th^ week of age, one group of rats had continued wheel access, while the other group had one week of wheel locking. Perirenal adipose tissue was extracted, RNA sequencing was performed, and bioinformatics analyses were executed using Ingenuity Pathway Analysis (IPA). IPA was chosen to assist in the understanding of complex ‘omics data by integrating data into networks and pathways. Wheel locked rats gained significantly more fat mass and significantly increased body fat percentage between weeks 10–11 despite having decreased food intake, as compared to rats with continued wheel access. IPA identified 646 known transcripts differentially expressed (p < 0.05) between continued wheel access and wheel locking. In wheel locked rats, IPA revealed enrichment of transcripts for the following functions: extracellular matrix, macrophage infiltration, immunity, and pro-inflammatory. These findings suggest that increases in visceral adipose tissue that accompanies the cessation of pubertal physical activity are associated with the alteration of multiple pathways, some of which may potentiate the development of pubertal obesity and obesity-associated systemic low-grade inflammation that occurs later in life.

## Introduction

The U.S. Centers for Disease Control and Prevention has reported that the overall prevalence of obesity among U.S. youth remains high (16.9%). Obesity is due to a positive caloric balance (intake > expenditure). Here we sought to understand transcriptomic changes underlying adipose tissue weight gain after the cessation of exercise. To address questions surrounding the influence of decreased energy expenditure on early obesity development in physically-active animals, our laboratory developed a unique polygenic model by which rats are provided voluntary physical activity (access to wheel running) for a given period of time, which is immediately followed by days of no physical activity by locking running wheels [wheel lock (LOCK)] [1–4]. In this model, young rats with continuous access to a voluntary running wheel exhibit lower intra-abdominal adipose tissue levels than rats without wheels. Upon cessation of voluntary running by LOCK, three sequential caloric events occur [1, 2]: 1) an inferred, decreased energy expenditure from cessation of voluntary running; 2) a rapid 2–3 day decrease in *ad libitum* caloric intake from rats with continued free wheel access (RUN); and 3) a rapid increase in intra-abdominal adipose tissue mass to levels of age-matched sedentary rats.

The unexpected finding from these earlier studies was the relative rapidity of increased visceral adipose tissue despite decreased energy expenditure simultaneous with decreasing caloric intake. Specifically, following 21 days of voluntary running in prepubertal rats (49–51 days of age), within a 2-day period of LOCK [1], we noted 30% and 48% increases in epididymal and omental adipose tissues, respectively. To gain greater insight into these unexpected findings, our second study used a longer period of voluntary running and a longer LOCK duration. Three week (wk) old rats were given access to voluntary running wheels for 6 wks, so that they were running ~9 km/day in the last week of running, after which wheels were locked during the 7^th^ week for 1 wk [2]. Confirming our previous results, epididymal, perirenal, and retroperitoneal adipose tissue masses increased 50%, 87%, and 100%, respectively, after 1 wk of LOCK. Based upon these results, we wanted to gain insight into potential mechanisms for the effects noted above. Therefore, the purpose of the current study was to employ unbiased transcriptomics to determine which cellular and metabolic pathways might be altered during LOCK, and thus may provide insight into the initial mechanisms by which obesity is facilitated, when food intake is paradoxically falling to help in explaining the rapid increase in perirenal adipose tissue (PRAT). Due to the short 7-day duration of LOCK causing a rapid increase in adipose tissue enlargement, previously observed by our lab using this model [1, 2], we hypothesized that we would observe increased transcripts for immune, inflammatory, or cell proliferation pathways due to the rapid increase in adipose tissue. Nishimura et al. [5] found that it took 6 and 10 wks before macrophage and CD8^+^ T cells, respectively, increased in the adipose stroma during development of obesity in diet-induced mice [5]. This hypothesis was generated based on the timeline of inflammatory changes being parallel to the increase in weight in the diet-induced obesity (DIO) model.

## Methods

### Animals and Experimental Design

The University of Missouri Animal Care and Use Committee approved all experimental protocols. Twelve male, Wistar rats were randomly selected from the 10^th^ generation population bred for high voluntary running distances in our laboratory [6]. Given previously observed rapid gains in fat mass following LOCK in high voluntary running rats [1, 2], we used this model to determine the transcriptomic alterations associated with this rapid fat gain. The rats were weaned at 21 days of age and then housed as 3–6 in a single cage, maintained on a 12-h light (07.00 h-19.00 h), 12-h dark (19.00–7.00 h) cycle, housed in the same temperature-controlled animal room (21°C), and provided *ad libitum* access to water and standard rodent chow (Formulab 5008, Purina Mills, St. Louis, MO, USA). At 28 days of age, 12 rats were relocated to individual cages equipped with voluntary running wheels and Sigma Sport BC 800 bicycle computers (Cherry Creek Cyclery, Foster Falls, VA), where they were housed for the remainder of the experiment. Running time and distance were recorded daily. Animal cages were exchanged with clean cages every 7 days at which time weekly food intake and body weight were recorded.

At 10 wks of age, one cohort of rats (RUN) maintained continued access to a voluntary running wheel from the 10^th^ to 11^th^ wk of age, while a second cohort of rats (LOCK), had their running wheels locked as to prevent voluntary wheel running for the last week of the study. Wheel running was selected because rats run further distances in wheels than in forced treadmill testing. No differences in wheel running distance or time were present between RUN and LOCK before beginning LOCK (RUN: 11.6 ± 2.3 km/day vs. LOCK: 11.0 ± 1.5 km/day), ensuring exposure to voluntary wheel running was similar between groups at 10 wks of age (data not shown). Further, RUN ran 10.7 ± 2.5 km/day during the final week of the study. The aim of the study was to compare PRAT transcriptomics between age-matched RUN and LOCK groups.

### Body composition analysis using DEXA

At 10 and 11 wks of age, body weight and composition were measured between 10.00 and 12.00, using a dual-energy X-ray absorptiometry (DEXA) machine calibrated for rats (QDR 4500A, Hologic, Inc. Bedford, MA). The DEXA scan at 10 wks was performed after brief anesthetization with isoflurane. At 11 wks of age, the DEXA scan was performed after CO_2_ asphyxiation and immediately prior to tissue removal.

### Animal sacrifice and tissue collection

Rats were sacrificed by CO_2_ asphyxiation between 10.00 and 12.00 as described previously [4]. The omental adipose tissue (OMAT), epididymal adipose tissue (EAT), and PRAT depots were removed, weighed, and parts of depots were used for either adipocyte sizing or snap-frozen in liquid nitrogen and stored at -80°C for mRNA analysis. However, only PRAT underwent RNA-seq analysis due to funding restrictions.

### Adipocyte size and number

At the time of sacrifice, three 60–80 mg fresh adipose tissue fragments from PRAT were weighed and placed in a glass scintillation tube with 3 ml Krebs-Ringer-HEPES buffer [(KRBH) 130 mM NaCl, 4.7 mM KCl, 1.2 mM KH_2_PO_4_, 10 mM HEPES, 1 mM CaCL_2_, 1.2 mM MgSO_4_, 0.25 mg/ml free fatty acid-free BSA, pH = 7.4]. Fragments were minced into ~1 mm^3^ pieces, incubated with 0.920 Wunsch units of Liberase Blendzyme (Roche) in a 37°C incubator at 100 rpm for 1 hour, transferred through a sterile 200-μm nylon mesh (Sefar America, Kansas City, MO) into a 15-ml conical tube, and centrifuged at 100 rpm for 1 minute to allow adipocytes to loosely collect at the top of the liquid. The bottom layer of liquid was removed using a syringe with needle and the adipocytes were washed with ~5 ml KRBH, after which they were then resuspended in 5-ml KRBH for every 1 ml of loosely packed adipocytes. A random sample of adipocytes was viewed with a Nikon Eclipse E600 microscope and photographed by an Olympus DP72 camera. Diameters for >300 adipocytes were measured using DP2-BSW v2.1 software. The mean diameter and size distribution were calculated for each adipocyte depot. The number of adipocytes per depot was estimated from the following calculation: the average cell mass was calculated by: average cell size (pl) x 0.915 (ng lipid/1 pl) x g/10^9^ ng; the number of cells per depot was calculated as: mass of adipose depot (g) / average mass of adipocyte (g). Reliability of this method is described in detail [7] and has been used by others [8].

### RNA isolation and preparation for conversion to cDNA for sequencing

The perirenal fat depot was selected for RNA-seq analysis because it is representative of visceral fat and exhibited dynamic increases during LOCK in the current study. Approximately 100 mg of frozen PRAT was homogenized in 1 ml RNA lysis buffer (Tri Reagent, Sigma, St. Louis, MO) containing stainless steel beads using a high speed-shaking device at 30 Hz for 1 min (Tissuelyser LT, Qiagen, Valencia, CA). RNA was isolated using manufacturer’s instructions (Tri Reagent, Sigma, St. Louis, MO). RNA integrity was checked using BioAnalyzer 2100 (Agilent Technologies Inc., Santa Clara, CA) automated electrophoresis system (Bio-Rad, Hercules, CA) prior to cDNA library construction at the University of Missouri’s DNA Core.

The cDNA library was constructed at the University of Missouri’s DNA Core using manufacturer’s protocol with reagents from Illumina’s TruSeq RNA sample preparation kit v2 (Illumina, San Diego, CA). Briefly, 1) the poly-A containing mRNA was purified from total RNA; 2) RNA was fragmented; 3) cDNA was generated from this fragmented RNA; 4) sample identifier adapters were ligated to the ends of cDNA; 5) the final constructs were evaluated using the BioAnalyzer 2100 automated electrophoresis system; 6) these constructs were quantified with the Qubit fluorometer (Invitrogen, Carlsbad, CA) using the quant-iT HS dsDNA reagent kit (Invitrogen); and 7) the solution was diluted for usage in HiSeq2000 (Illumina, San Diego, CA) employing Illumina’s standard sequencing protocol.

RNA-seq procedures were performed at the University of Missouri’s DNA Core and performed, as described elsewhere [9]. Briefly, 1) samples were loaded into a flowcell where each olgionucleotide was replicated; 2) flowcells were placed in the sequencer and fluorescently labeled bases were attached to each complementary base; 3) 50-base pair (bp) reads were recorded with the Illumina Genome Analyzer (Illumina, San Diego, CA); 4) adaptor sequences reads were trimmed; 5) reads were tiled to a custom database consisting of rat and human homologous sequences using NexGen v2.2 software (SoftGenetics, State College, PA); and 6) Reads per kilobase per million reads (RPKM) values were assigned to each transcript. RPKM is a value that quantifies gene expression from DNA sequencing data that normalizes for the number of sequencing reads and total read length [10] and is the value that is used to compare transcript expression among groups.

### Transcript filtering process and bioinformatics analysis

**Fig 1:**
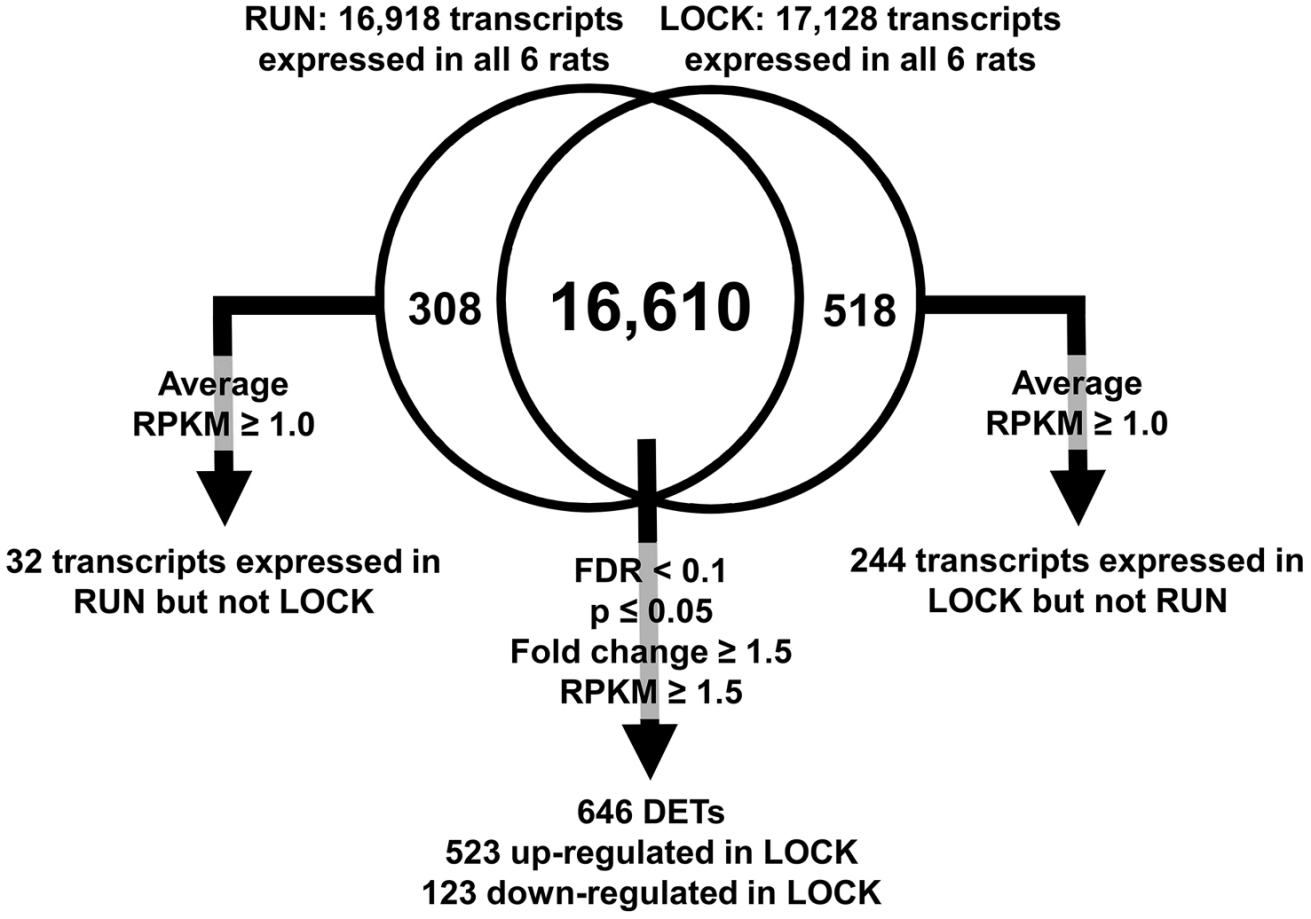
Transcript filtering after RNA-sequencing. For each group, a transcript was omitted if it was not a known transcript or was not expressed in one experimental group. This filtering produced 16,918 and 17,128 known transcripts in the continued wheel access (RUN) and wheel locking (LOCK) groups, respectively. Between the RUN and LOCK groups, there were 16,610 commonly expressed genes that were compared and filtered for significance. A transcript was deemed significantly different between RUN and LOCK if: 1) false discover rate (FDR) < 0.1, 2) p<0.05, 3) fold change ≥ 1.5, and 4) average RPKM ≥ 1.5, 646 transcripts met these criteria. Of these 646 transcripts, 523 were up-regulated and 123 were down-regulated in LOCK, respectively. Abbreviations: Reads Per Kilobase Per Million Reads (RPKM); and Differentially Expressed Transcripts (DETs).

Currently, there is no universally accepted standard for RNA-seq bioinformatics, therefore the current RNA-seq literature was examined and we devised an approach for a modified filtering process that incorporated aspects of other current filtering procedures [6, 11, 12] into the dataset. All bioinformatics procedures were performed with Microsoft Excel v2007 (Redmond, WA). The filtering process is described in the Fig 1 legend. Ingenuity Pathway Analysis (IPA; Ingenuity Systems, Inc., Redwood, CA) was used to identify uniquely up- and down-regulated networks and pathways with LOCK. The complete filtered data set is presented in S1 Table. Transcripts present in only RUN or LOCK are presented in S2 and S3 Tables, respectively.

### Western blotting

Western blot analysis was performed for the determination of the protein content of mature Col1a1 (sc-8784, Santa Cruz Biotechnology, Santa Cruz, CA) and CD36 (ab133625, Abcam, Cambridge, MA), as previously described [13]. To control for equal protein loading and transfer, membranes were visualized with Ponceau S as previously described [13]. All data are expressed in arbitrary units.

### qRT-PCR validation of RNA-seq

For quantitative real-time polymerase chain reaction (qRT-PCR), 1μg of RNA from PRAT samples was reverse transcribed using a High Capacity cDNA Reverse Transcriptase kit (Applied Biosystems, Carlsbad, CA). Primers for specific genes were constructed (Table 1) using PrimerExpress 3.0 software (Applied Biosystems). Twenty-five nanograms of cDNA from each sample were assayed in duplicate for the target genes listed in Table 1 using SYBR Green Mastermix (Power SYBR green Mastermix, Applied Biosystems). mRNA expression values were determined using the 2^ΔΔCt^ method, whereby ΔCT = 18S Ct − gene of interest Ct. mRNA expression values were normalized to RUN values.

**Table 1:** Primer sequences for gene expression analyzed by qRT-PCR.

Gene	Forward (5’- 3’)	Reverse (5’- 3’)	Accession no.
18S	GCCGCTAGAGGTGAAATTCTTG	CATTCTTGGCAAATGCTTTCG	NR_046237.1
Col1a1	GACTGTCCCAACCCCCAAAA	CTTGGGTCCCTCGACTCCTA	NM_053304.1
Col3a1	GCTCGGAATTGCAGAGACCT	AGCATCCATCTTGCAGCCTT	NM_032085.1
Tlr2	ATCACTGCACCCTCAATGGG	TGTGCAGGCTCCGTATTGTT	NM_198769.2
Cd36	ACTCTCTCCTCGGATGGCTA	TGCATGAACAGCAGTATCTGAGT	NM_031561.2
Fabp3	GTCGGTACCTGGAAGCTAGTG	CTTGGTCATGCTAGCCACCT	NM_024162.1
Fabp4	CAGCGTAGAAGGGGACTTGG	TTCATCGAATTCCACGCCCA	NM_053365.1
Agpat9	AGACACTCAGGTCTGAGCCA	GCGAAGAGGAAAGGGGAGAC	XM_008770019
Cish	CAACACCTGTGTCGGCTAGT	AACGGGTACTGTCGGAGGTA	NM_031804.1

### Statistical analysis

Outcome measures for between-group comparisons of animal characteristics, (body mass, lean mass, fat mass, body fat percentage, and food intake), delta measurements (change in body weight, lean body mass, fat mass, and percent body fat), and tissue measurements (depot mass, adipocyte diameter, and total adipocyte number) were analyzed using Student’s t-test. Significant differences are noted with lower-case letters in tables and graphs. Sigmaplot 12.0 (San Jose, CA) was used for all statistical analyses. Values are reported as means ± SE, and significance was set at p < 0.05. Any exceptions to these statistical methods are stated in the figure legends.

## Results

### Morphological values and food intake

**Fig 2:**
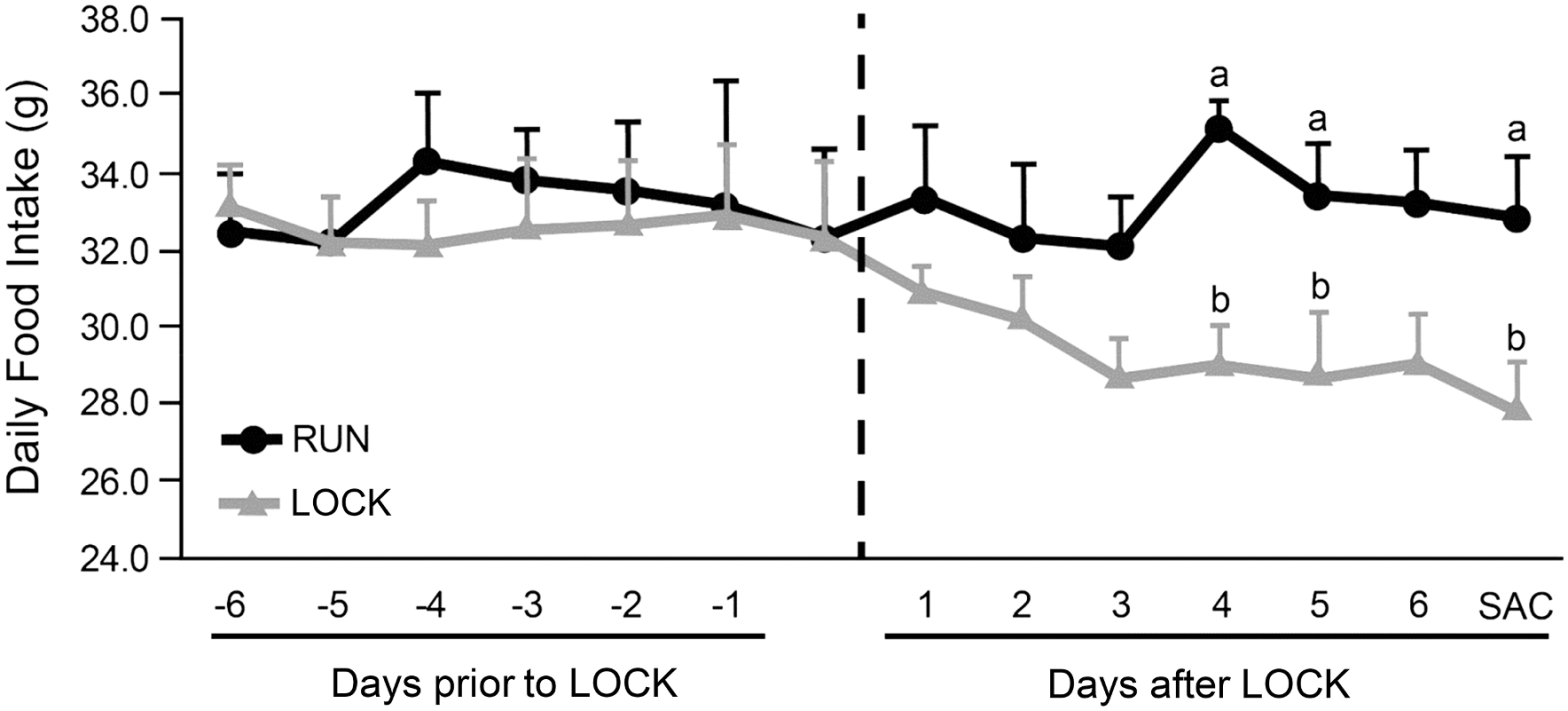
Daily food intake over the final 14 days. The vertical dashed line represents the day of wheel locking (LOCK). Different letters denote significance among groups at p < 0.05, as determined by Student’s t-test.

Animal characteristics are presented in Table 2. LOCK for 1 wk showed significantly greater increases in body mass (23.8 g vs. 14.3 g, p < 0.05), fat mass (9.1 g vs. -0.4 g, p < 0.05), and body fat percentage (2.0% vs. -0.7%, p < 0.05) when compared to RUN between 10 and 11 wks of age. PRAT mass was significantly greater (3.43 g vs. 1.93 g, p < 0.05) after 1-wk LOCK compared to RUN. Food intake was not significantly different between groups for the first 3 days of LOCK, but the LOCK group ate significantly less than RUN on days 4, 5 and 7 (p < 0.05) (Fig 2).

**Table 2:** Animal characteristics.

Measurements	RUN	LOCK
Body mass 10wk, g	350.5 ± 13.7	346.5 ± 3.6
Body mass 11wk, g	364.8 ±13.9	370.3 ± 4.0
Change in body mass (last 7 days), g	14.3 ± 0.6	23.8 ± 2.0
Lean mass 10wk, g	324.0 ± 10.0	320.5 ±4.4
Lean mass 11wk, g	338.6 ± 10.7	335.1 ± 5.4
Change in lean mass (last 7 days), g	14.7 ± 1.9	14.6 ± 2.3
Fat mass, 10wk, g	26.6 ± 4.9	26.0 ± 2.7
Fat mass, 11wk, g	26.2 ± 5.0	35.1 ± 4.0
Change in fat mass (last 7 days), g	-0.4 ± 1.9	9.1 ± 1.7 *
Perirenal fat mass, g	1.93 ± 0.26	3.43 ± 0.25 *
Perirenal adipocyte diameter, μm	62.2 ± 3.9	67.1 ± 1.4 *
Perirenal depot cellularity (x 10^6^)	14.9 ± 1.1	24.2 ± 2.9 *
Epididymal fat mass, g	2.96 ± .017	4.04 ± 0.44 *
Epididymal adipocyte diameter, μm	64.8 ± 3.6	68.3 ± 2.3
Epididymal depot cellularity (x 10^6^)	24.3 ± 3.4	26.3 ± 1.8
Omental fat mass, g	0.35 ± 0.02	0.47 ± 0.07 *
Omental adipocyte diameter, μm	51.7 ± 3.4	54.9 ± 2.4 *
Omental depot cellularity (x 10^6^)	5.9 ± 1.0	5.8 ± 0.5
Body fat % 10wk, g	7.4 ± 1.1	7.5 ± 0.8
Body fat % 11wk, g	7.0 ± 1.1	9.5 ± 1.1
Change in body fat % (last 7 days), g	-0.7 ± 0.4	2.0 ± 0.4 *

Student’s t-test was performed on each outcome measure for continued wheel access (RUN) and wheel locking (LOCK) groups. Values are means ± SE. n = 6 per group.

* denotes significant differences between groups at p<0.05.

### PRAT adipocyte size distributions

**Fig 3:**
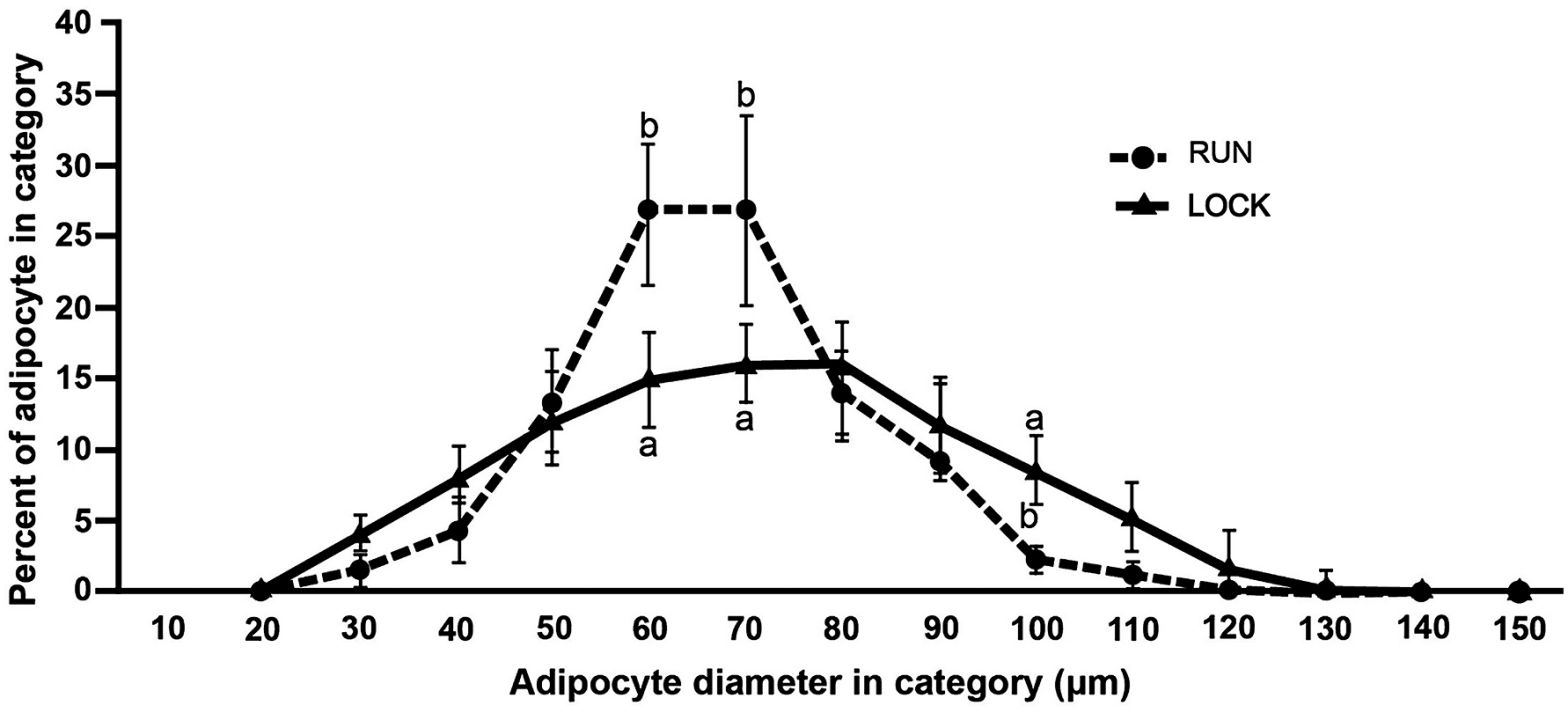
PRAT adipocyte size distribution. Adipocyte diameters were classified in 10-μm categories [category #, adipocyte diameter range in μm (10: 0.0–9.0μm, 20: 10.0–19.9μm, 20: 20.0–29.9μm, etc.] on the x-axis; and the percentage of total adipocytes in a 10-μm category was plotted on y-axis. At least 300 adipocyte diameters were measured for each tissue sample. Different letters denote differences among groups at each 10-μm category, p<0.05, as determined by Student’s t-test.

PRAT adipocyte diameters were significantly greater in LOCK (67.1 ± 1.4) compared to RUN (62.2 ± 3.9) (p < 0.05); consequently, adipocyte cell number was estimated to be significantly increased in PRAT in LOCK compared to RUN (24.2 x 10^6^ vs 14.9 x 10^6^, p < 0.05). However, RUN adipocyte diameters were smaller for the range of 50 to 69.9 μm than in LOCK (Table 2). The peak of the adipocyte size distribution curve for the LOCK group was flatter and lower when compared to RUN, with significant differences in adipocyte size distribution occurring between 50.0–69.9, 60.0–79.9, and 90.0–99.9 μm in diameter (Fig 3).

### Transcript differences between RUN and LOCK

The overall numbers of known transcripts expressed in RUN and LOCK are shown in Fig 1, and its legend. There were 16,918 RUN and 17,128 LOCK known transcripts expressed. Of these transcripts, 16,610 were commonly expressed between RUN and LOCK groups that were filtered and compared for analysis. From our filtering process, 646 transcripts were differentially expressed in LOCK compared to RUN (523 upregulated in LOCK, and 123 down-regulated in LOCK). Of note, cyclin A1 was down-regulated in RUN rats (fold change = -1.72, p = 0.0016), confirming our RT-PCR outcome in our earlier publication [4]. The top 15 up-regulated and down-regulated (fold change in LOCK compared to RUN) mRNAs in PRAT of LOCK rats are shown in Table 3. We also identified 32 transcripts expressed in RUN but not LOCK, and 244 transcripts expressed in LOCK but not RUN. The top 10 up-regulated and down-regulated mRNAs present in either RUN or LOCK are shown in Tables 4 and 5, respectively.

**Table 3:** Top 15 up and down regulated genes differentially expressed between continued wheel access (RUN) and wheel locking (LOCK), sorted by magnitude of fold change.

Gene Transcript	Description	LOCK/RUN	RUN Average RPKM	LOCK Average RPKM
*Upregulated with LOCK*				
RETNLG	Resistin-like gamma	7.400	3.93	29.08
CLEC11A	C-type lectin domain family 11, member A	5.522	2.09	11.52
LOX	Lysyl oxidase	4.549	8.567	38.97
SFRP1	Secreted frizzled-related protein 1	4.281	4.221	18.08
PTGIS	Protaglandin I2 synthase	4.065	8.745	35.55
FLRT2	Fibronectin leucine rich transmembrane protein 2	3.987	2.778	11.08
CCL13	Chemokine (C-C motif) ligand 13	3.630	9.931	36.05
MT2A	Metallothionein 2A	3.488	3.772	13.16
LUM	Lumican	3.467	42.351	146.95
C3	Complement component 3	3.429	3.256	11.17
FN1	Fibronectin 1	3.396	59.75	202.91
KIAA0101	Kiaa0101	3.382	2.80	9.47
SPON1	Spondin 1	3.355	7.99	26.80
ELN	Elastin	3.296	35.10	115.71
LYVE1	Lymphatic vessel endothelial hyaluronan receptor 1	3.286	20.73	68.15
*Downregulated with LOCK*				
SYTL5	Synaptotagmin-like 5	-2.778	5.96	2.15
FABP3	Fatty acid binding protein 3	-2.740	17.87	6.52
CISH	Cytokine inducible SH2-containing protein	-2.470	30.12	12.19
TMEFF1	Transmembrane protein with EGF-like and two follistatin-like domains 1	-2.456	15.11	6.15
CALML3	Calmodulin-like 3	-2.377	17.35	7.30
ADHFE1	Alcohol dehydrogenase, iron containing, 1	-2.359	8.97	3.80
HOXA9	Homeobox A9	-2.288	49.27	21.54
SIX1	SIX homeobox 1	-2.245	82.30	36.58
UCP3	Uncoupling protein 3	-2.221	6.56	2.95
KIFC2	Kinesin family member 2	-2.151	8.46	3.93
CYP4F22	Cytochrome P450, family 4, subfamily V, polypeptide 22	-2.067	16.68	8.07
TBATA	Thymus, brain and testes associated	-2.043	6.96	3.41
NABP1	Nucleic acid binding protein 1	-1.980	106.20	53.64
HOXC10	Homeobox C10	-1.973	5.86	2.97
RESP18	Regulated endocrine-specific protein 18	-1.952	7.38	3.78

**Table 4:** Top 10 genes expressed in RUN but not LOCK, sorted by average RPKM.

Gene Transcript	Description	RUN RPKM
RFX4	Regulatory factor X, 4	2.44
HEY2	Hairy/enhancer-of-split related with YRPW motif 2	2.53
GIPR	Gastric inhibitory polypeptide receptor	2.10
TSHR	Thyroid stimulating hormone receptor	2.01
GRTP1	Growth hormone regulated TBC protein 1	1.86
NCKAP5	NCK-associated protein 5	1.75
ACE2	Angiotensin I converting enzyme (peptidyl-dipeptidase A) 2	1.67
CLMP	CXADR-like membrane protein	1.60
PDE4C	Phosphodiesterase 4C, cAMP-specific	1.52
NEU2	Sialidase 2 (cytosolic sialidase)	1.48

**Table 5:** Top 10 genes expressed in LOCK but not RUN, sorted by average RPKM.

Gene Transcript	Description	LOCK RPKM
TOP2A	Topoisomerase (DNA) II alpha	7.42
CDKN3	Cyclin-dependent kinase inhibitor 3	7.13
MK167	Antigen identified by monoclonal antibody Ki-67	5.87
TPX2	TPX2, microtubule-associated, homolog	5.11
NUSAP1	Nucleolar and spindle associated protein 1	5.01
CCNA2	Cyclin A2	4.85
RACGAP1	Rac GTPase activating protein 1	4.58
CKAP2	Cytoskeleton associated protein 2	4.50
AURKB	Aurora kinase B	4.32
KIF23	Kinesin family member 23	4.16

### IPA network analysis

Ingenuity Pathway Analysis (IPA) was used to generate hypotheses after our unbiased collection of differentially expressed transcripts that were obtained with global transcriptomics. IPA provides estimates of pathways altered by experimental treatments. Two top scoring IPA networks that were inclusive for both up-regulated and down-regulated mRNAs from LOCK rats had scores of 41 and included 34 and 35 transcripts, respectively (Table 4). Using an algorithm that aggregates genes in our dataset based on existing pathway relationships in the IPA knowledge base, in one network, IPA identified the following functions: “cancer,” “organismal injury and abnormalities,” and “reproductive system disease.” Of the top 15 up- and down-regulated transcripts, SFRP1 (up-regulated), KIAA0101 (up-regulated), and HOXA9 (down-regulated) were involved with the network (Table 6). The other top scoring network had the following functions: “lipid metabolism,” “small molecule biochemistry,” and “cellular development.”

**Table 6:** List of transcripts from top scoring IPA networks inclusive of both up-regulated and down-regulated genes as influenced by wheel locking (LOCK) (score = 41 for both networks).

Network: Cancer, organismal injury and abnormalities, and reproductive system disease (34 mRNAs)
*Up-regulated in LOCK*
Anxa2, Arhgap11a, Cd34, Cdca4, Celf2, Dnmt1, E2f1, Elf4, Fkpb10, H1fx, H2afx, Hmgb2, Mcm2, Mcm4, Mcm6, Nasp, Ndn, Orai2, Pcna, Pold3, Smoc2, Soga1, Spats2l, Wee1
*Down-regulated in LOCK*
Cyc1, Hoxa9, Hspe1
*Non-changing*
Cd3, Cdc2, Cyclin A, E2f, Histone h1, Histone h3, Histone h4
Network: Lipid metabolism, small molecule biochemistry, cellular development (35 mRNAs)
*Up-regulated in LOCK*
Alcam, Aldh1a3, Alox5ap, B3gnt9, Card6, Coro1a, Dmbt1, Glrx, Lama2, Ltc4s, Minos1-nbl1/nbl1, Myo1e, Rab31, Rab3d, Rin3, Selplg, Soat1, Stk10, Tifa, Tpm3, Trafd1, Vnm1
*Down-regulated in LOCK*
Cdkn2c, G0s2, Hmgcs1, Insig1, Phyh
*Non-changing*
Amylase, Bmp, Elastase, Ets, Fascin, Hmg CoA synthase, Igh (family), Nfkb (complex)

**Fig 4:**
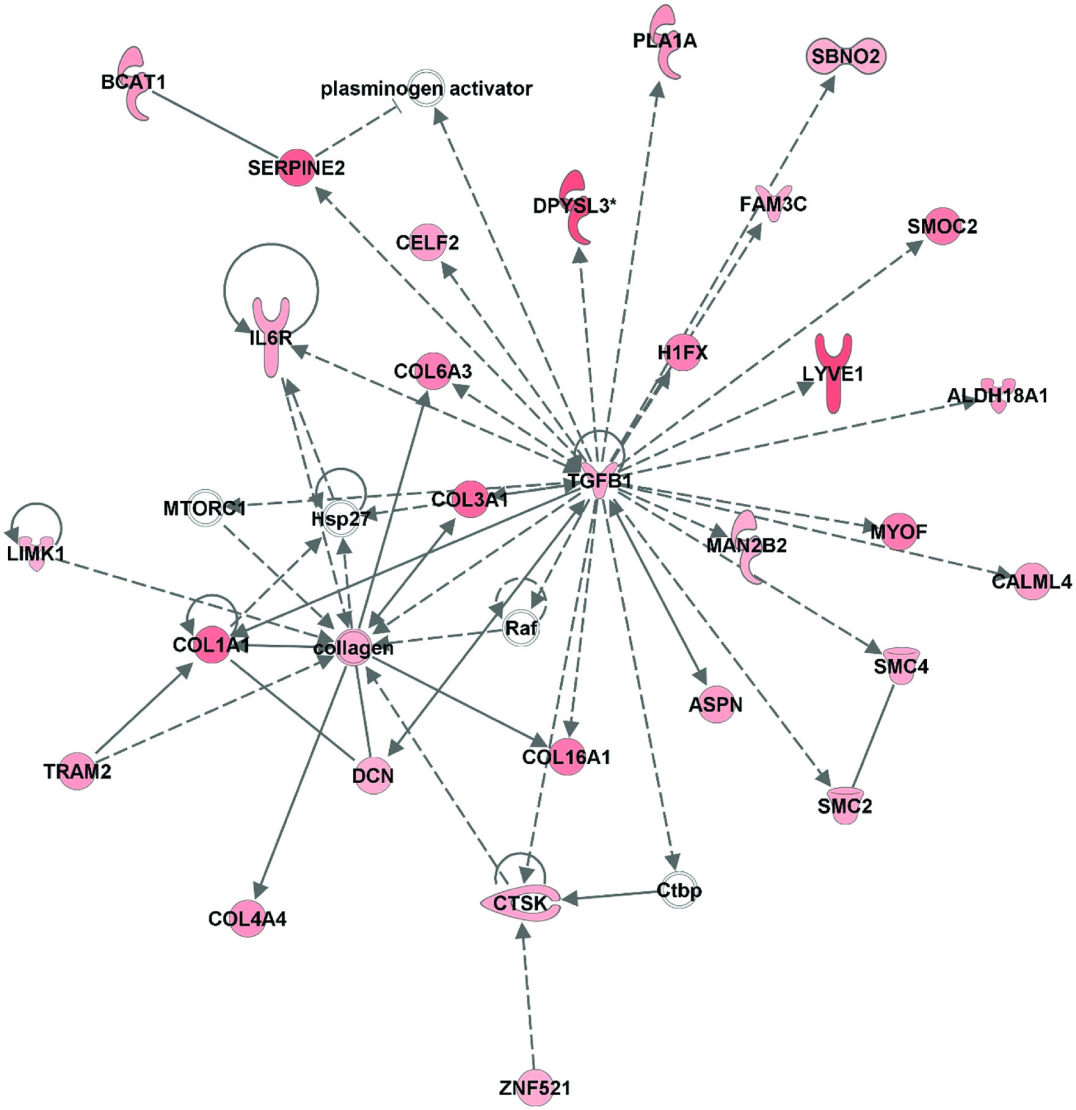
Top scoring network inclusive of up-regulated genes (score = 47) in LOCK vs RUN. Nodes represent genes/molecules. Shading is proportional to fold change size (red: up-regulated). Direct and indirect relationships are denoted with solid and dashed lines, respectively. White nodes denote network members that were not altered in the network. Lines ending in an arrow or blunt end indicate known direction of molecular activation or inhibition, respectively. Different shapes of genes represent different gene functions.

**Fig 5:**
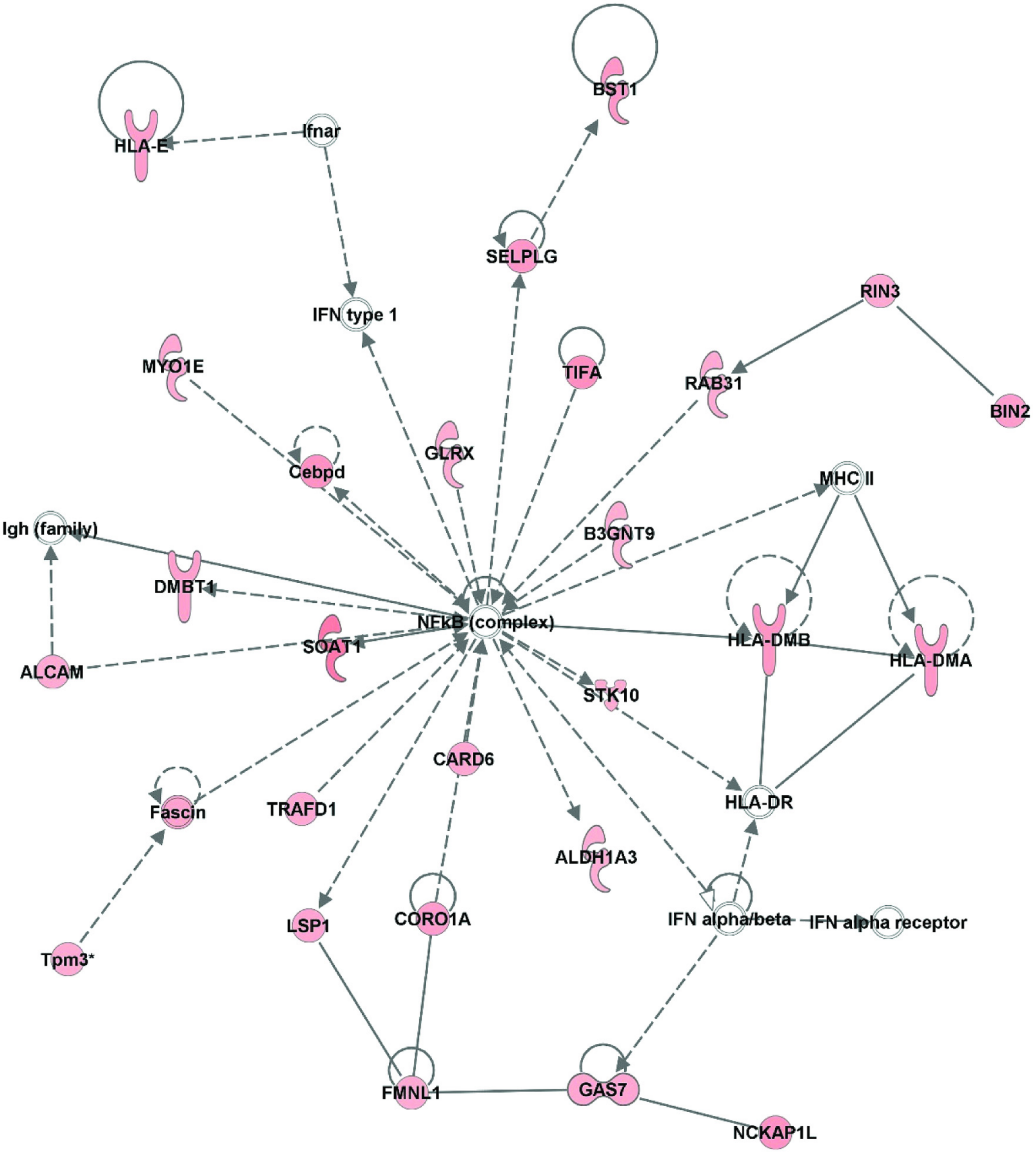
Second scoring network inclusive of up-regulated genes (score = 40) in LOCK vs RUN. Nodes represent genes/molecules. Shading is proportional to fold change size (red: upregulated). Direct and indirect relationships are denoted with solid and dashed lines, respectively. White nodes denote network members that were not altered in the network. Lines ending in an arrow or blunt end indicate known direction of molecular activation or inhibition, respectively. Different shapes of genes represent different gene functions.

The top scoring IPA network inclusive of only up-regulated mRNAs in LOCK had a score of 47 and included 35 transcripts. IPA identified top functions for this network as “organismal injury and abnormalities,” “gastrointestinal disease,” and “dermatological diseases and conditions.” 29 of the 35 mRNAs in this pathway were up-regulated (6 were not up-regulated), and many of these 29 transcripts had direct interactions with TGF-β1 (Fig 4). Similarly, the second scoring network inclusive of up-regulated mRNAs in LOCK had a score of 40, included 34 transcripts (26 up-regulated), and had top functions identified by IPA as “cell morphology,” “hematological system development and function,” and “inflammatory response.” Many of the up-regulated transcripts had direct interaction with the NF-κB complex (Fig 5). The top scoring IPA network containing only down-regulated mRNAs from LOCK rats had a score of 58 and included 35 mRNAs (26 down-regulated) (Table 7), and the top functions identified by IPA included “metabolic disease,” “lipid metabolism,” and “molecular transport.”

**Table 7:** List of transcripts from top scoring IPA network inclusive of only down-regulated genes as influenced by wheel locking (LOCK) (score = 58).

Network: Metabolic disease, lipid metabolism, molecular transport (35 mRNAs)
*Down-regulated in LOCK*
Acox1, Acsl1, Adipor2, Agpat9, Aig1, Alas1, Asns, Cd36, Ces1, Ces1d, Ces1e, Cntf, Dnajc15, Fabp4, Fdps, G0s2, Gpd1, Hmgcs1, Hspe1, Insig1, Kcnh2, Mvd, Oxct1, Pxmp2, Retsat, S100a1
*Non-changing*
Fascin, Hla-dr, Ifn alpha receptor, Ifn alpha/beta, Ifn type 1, Ifnar, Igh (family), Mhc II, Nfkb (complex)

**Fig 6:**
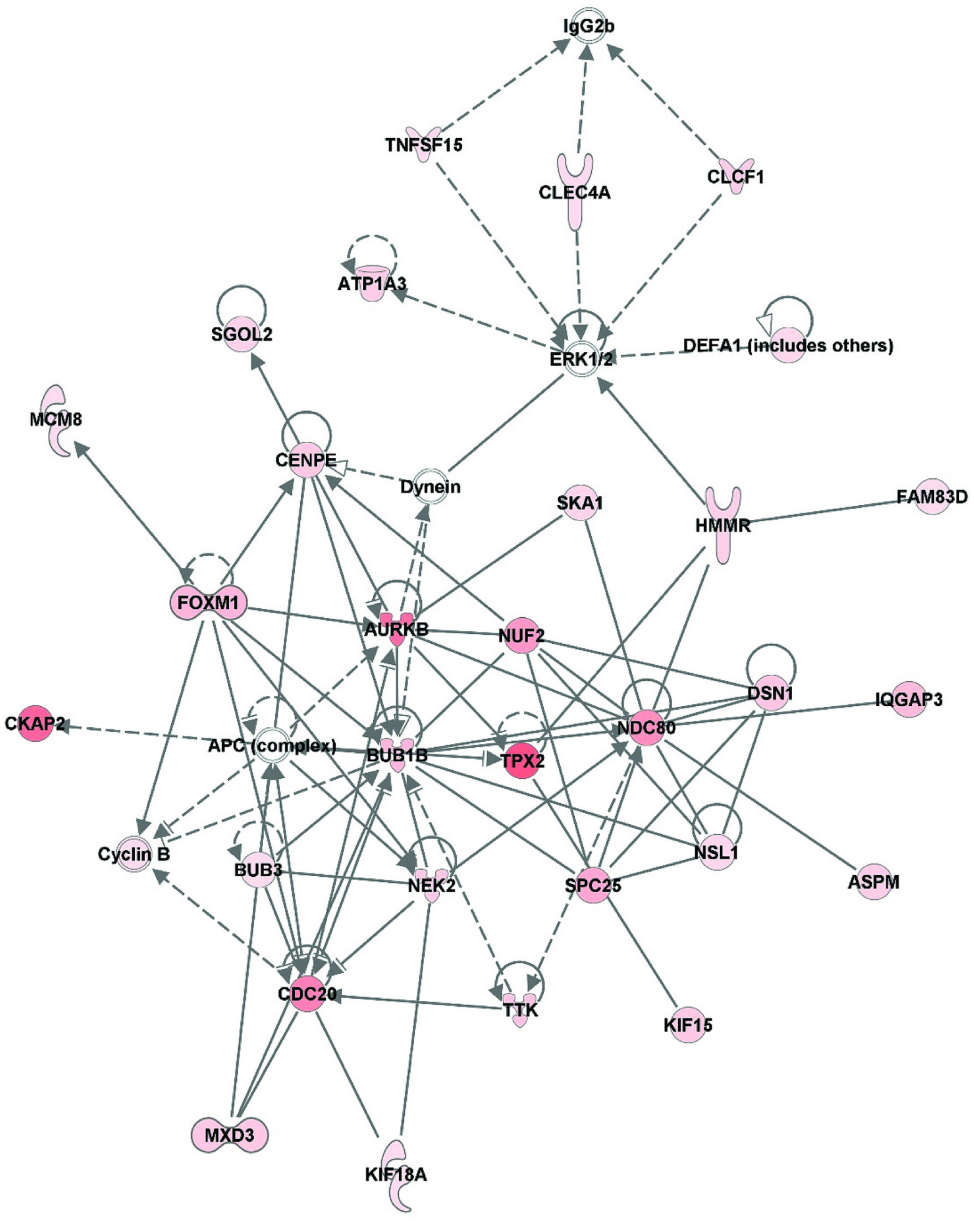
Top scoring network inclusive of genes expressed only in LOCK (score = 63). Nodes represent genes/molecules. Shading is proportional to RPKM value. Direct and indirect relationships are denoted with solid and dashed lines, respectively. White nodes denote network members that were not altered in the network. Lines ending in an arrow or blunt end indicate known direction of molecular activation or inhibition, respectively. Different shapes of genes represent different gene functions.

For analysis of the 244 transcripts expressed exclusively in LOCK, the top scoring IPA network had a score of 63 and included 35 transcripts, with 30 mRNAs specific to LOCK. IPA identified top functions for this network as “cell cycle,” “cellular assembly and organization,”“DNA replication,” “recombination,” and “repair.” (Fig 6). Of the top 10 transcripts expressed, only LOCK, TPX2, CKAP2, and AURKB were involved with the network. In analysis exclusive to transcripts expressed only in RUN, the top scoring IPA network had a score of 27 and included 35 transcripts, with 11 mRNAs specific to RUN, with the functions “developmental disorder, endocrine system disorders, metabolic disease” (data not shown).

### IPA predicted diseases and common targets associated with LOCK

Given the association between visceral adipose tissue enlargement and disease progression, we assessed the top diseases and disorders predicted by IPA to be increasing with LOCK. Further, we sought to identify transcripts common to several top predicted diseases to begin to identify targets that may associate with the detrimental effects of LOCK. Three of the top diseases and disorders predicted by IPA to associate with LOCK included “inflammatory response,” “vascular disease,” and “cancer.” We used the three aforementioned diseases/conditions to search for whether they had a common transcript(s). Our rationale was to discover common transcripts, and then generate new hypotheses common to these diseases/disorders. In an effort to identify targets associated with these disorders, we identified 167 out of 646 differentially expressed transcripts that were associated with at least one of the included diseases/conditions. We next filtered this list to identify the transcripts common to all three diseases/conditions (inflammatory response, vascular disease, cancer) and found 12 out of 646 differentially expressed transcripts. Finally, this list was trimmed to include only transcripts with expression occurrences matching the disease/condition pathway. In doing so we identified 5 transcripts common to inflammatory response, vascular disease, cancer associated pathways: fibronectin 1 (Fn1) (fold-change LOCK/RUN = 3.4), cytochrome b-245, beta (Cybb) (fold-change LOCK/RUN = 2.4), protein kinase C, beta (Prkcb) (fold-change LOCK/RUN = 1.9), iron-trafficking protein lipocalin 2 (Lcn2) (fold-change LOCK/RUN = 1.7), and transcription-activator protein Stat3 (fold-change LOCK/RUN = 1.6). From this, we speculate the 5 highlighted transcripts may play some role to many pathological changes in adipose tissue associated with LOCK.

### Validation of RNA-seq by qRT-PCR

Based upon IPA analysis, we analyzed 8 mRNAs for validation of RNA-seq. Comparisons of qRT-PCR and RNA-seq results are presented in Table 6. We observed confirmatory increases in Col1a1, Col3a1, and Tlr2, and decreases in Cd36, Fabp3, Fabp4, Agpat9, and Cish in LOCK compared to RUN (p < 0.05) (Table 8).

**Table 8:** Validation of select RNA-seq transcripts by qRT-PCR.

	RNA-seq		qRT-PCR	
Transcript name	Fold change	P-value	Fold change	P-value
Col1a1	2.73	0.008	1.83	< 0.001
Col3a1	2.74	0.019	1.78	< 0.001
Tlr2	2.09	0.029	1.34	0.049
Cd36	-1.53	< 0.001	-1.49	0.003
Fabp3	-2.74	0.012	-1.76	0.020
Fabp4	-1.73	< 0.001	-1.25	0.018
Agpat9	-1.68	< 0.001	-1.49	0.005
Cish	-2.47	0.047	-2.43	0.049

### Protein expression

**Fig 7:**
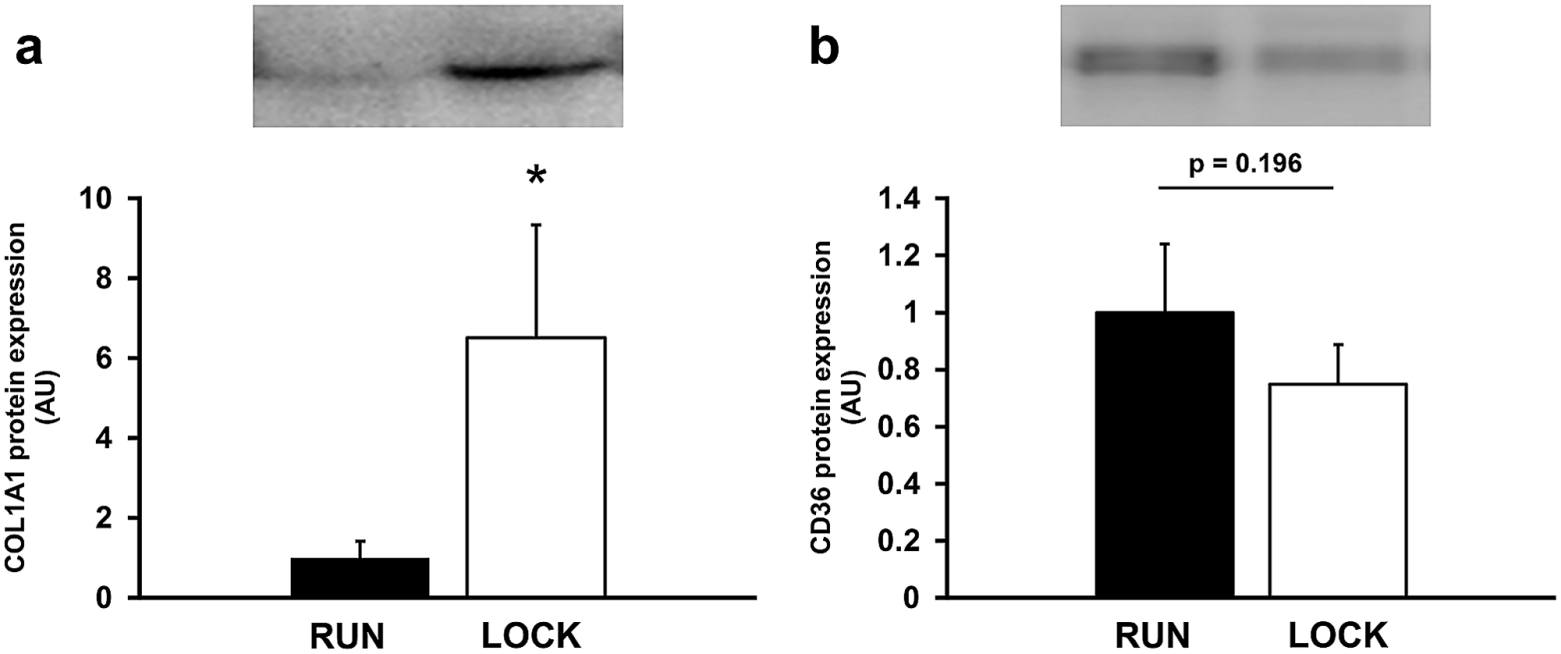
Protein expression of A) Col1a1 and B) Cd36 in PRAT from continued wheel access (RUN) and wheel locking (LOCK) rats (n = 5 per group). Protein expression was normalized for Ponceau S.

To further validate our top up- and down-regulated transcripts, we performed Western blot analysis on two molecules with central functions as identified by IPA. Western blot showed mature COL1A1 protein was increased ~6-fold in LOCK compared to RUN (p = 0.045) and CD36 protein was not different between groups (p = 0.196) (n = 5/group) (Fig 7). Thus, qRT-PCR detected a significant decrease in CD36 mRNA, while only a non-significant trend was observed in the Western blot of CD36 protein in Fig 7. One possible explanation could be CD36 protein had a larger standard error.

## Discussion

Herein, we report for the first time the rapid genome-wide transcriptomic changes in PRAT that accompany a one week loss of daily physical activity using the LOCK model. Furthermore, it is important to note that LOCK-induced increases in PRAT mass occurred despite decreased food intake over this period. In diet-induced obesity models, rats and mice fed high fat-diet require weeks to exhibit overt obesity [14–16]. Thus, the current findings clearly and importantly illustrate that early transcriptomic disturbances accompany rapid increases in PRAT diameter and cellularity following only 1 week of LOCK, as opposed to the aforementioned rodent diet-induced obesity models. The observation of rapid transcriptomic changes makes this model a very powerful tool to study the etiology of physical inactivity-induced increases in visceral adiposity.

Consistent with our earlier findings [1, 2], where we observed rapid enlargement of visceral adipose tissue after 2 and 7 days of LOCK as well as an increase in body fat percentage, we noted an increase in PRAT mass and body fat percentage in this study. Expanding upon these morphological findings, we performed an unbiased approach using RNA-seq to uncover transcriptome-wide changes that accompany the rapid calorie-intake independent, physical inactivity-induced increase in a visceral fat pad mass.

Of the 646 differentially expressed transcripts identified, we observed ~4.2-fold greater up-regulated than down-regulated transcripts. One possible explanation of this observation is that increased activation/up-regulation is due to increased adipocyte proliferation and expansion. Given this difference, we first analyzed networks inclusive of upregulated mRNAs. Several of the functions of the top-scoring IPA networks (inclusive of up-regulated mRNAs) include various types of “disease” or “injury” including gastrointestinal, dermatological, and organismal, as well as “cell morphology” and “inflammatory response.” The functional implications of the aforementioned IPA networks imply that PRAT after 7 days of LOCK is undergoing rapid changes in inflammatory and other signaling that are precursors to disease progression.

Increased expression of mRNAs involved in extracellular matrix (ECM) remodeling, proliferation, and differentiation have been reported with adipose tissue development at the onset of diet-induced obesity [17, 18]. Consistent with findings from the early-stages of diet-induced obesity studies, our network analysis identified increased expression of common collagen-related ECM transcripts COL1A1, COL3A1, COL16A1, COL4A4, and COL6A3. These changes were associated with an increase in TGFβ mRNA, whose protein has proliferation and differentiation functions [17]. In addition, transcripts involved in crosslinking such as lysyl oxidase (LOX) [19], fibronectin leucine rich transmembrane protein 2 (FLRT2) [20], fibronectin 1 (FN1) [21], lumican (LUM) [17], elastin (ELN) [17], were among the top 15 upregulated with 1 wk of LOCK. This up-regulation in fibrotic transcripts is in agreement with our observed adipocyte hypertrophy, as discussed previously [17], and is associated with increased local inflammation [22, 23]. Metallothionein 2A was increased ∼350%; its increase in human embryonic kidney (HEK) cells has been associated with a reduced level of cytochrome *c* oxidase subunit II protein, resulting in lower mitochondrial complex IV activity and reduced the oxidative phosphorylation capacity of HEK cells [24]. In a similar manner, hindlimb immobilization increases metallothionein mRNA and produces a threefold decrease transcripts for enzymes involved in energy metabolism [25].

ECM remodeling, as described in the above paragraph, is promoted by macrophage infiltration [26]. Remarkably, gene markers of macrophage infiltration had rapidly increased over a 7-d LOCK period. In addition, further analysis of unfiltered data revealed that transcript increases of 4.0-fold, 2.1-fold, and 2.5-fold were found for macrophage-inflammatory protein-1α [MIP-1α (involved in acute inflammation)], F4/80 (classical macrophage surface protein), and CD68 [macrosialin (expressed specifically in macrophages and macrophage-related cells)], respectively. In contrast to the LOCK data presented herein, a diet-induced obesity murine model reported no changes in these three aforementioned macrophage gene markers in epididymal adipose tissue until the 16^th^ wk of high-fat feeding [27].

We contend that the rapid LOCK-induced elevation in pro-inflammatory transcript markers that accompanies visceral adipose tissue enlargement is remarkably noteworthy. Weisberg at al. [28] estimated that macrophage percentage in adipose tissue ranges from <10% in lean mice and humans to >50% in extremely obese, leptin-deficient mice, and almost 40% in obese humans. Others have reported that when high-fat diets were fed to mice, CD8^+^CD4^-^ T cell infiltration precedes macrophage accumulation [5]. Specifically, high-fat feeding in the aforementioned study began at 4 wks of age, and within 2 wks of initiating high-fat feeding, increases in the CD8^+^CD4^-^ T cell fraction were present in the stroma of the epididymal fat [5], a timeline that seems somewhat rapid given the slower rate of fat gain in DIO studies. In contrast, these authors also reported that macrophages began to infiltrate adipose tissue by the 10^th^ wk of high-fat feeding. Similarly, others have reported that an increase in macrophage accumulation occurs by the 16^th^ wk of high-fat feeding [27]. One explanation for the differences between the current study and many DIO studies could lie in the rate of fat enlargement if inflammation is dependent upon the absolute mass of adipose tissue. Our data demonstrate that, not only does the cessation of routine physical activity lead to a rapid expansion of visceral adipose tissue, but it may lead to a rapid infiltration of macrophages which have the potential to drive a pro-inflammatory adipose tissue phenotype [29]. Collectively, the similar transcriptomic profiles between our 1 wk LOCK experiment and longer (10–16 wk) DIO studies suggest that the rapidity of fat gain may ultimately determine the timeframe for transcriptomic changes. Thus, the rapid transcriptomic changes, which we observe in LOCK are potentially the result of very rapid increases in fat mass compared to DIO models that often have a more delayed timeline for fat gain.

The final filtering from the 646 differently expressed transcripts retained five transcripts to increase with LOCK that are common to three highly predicted diseases/conditions: 1) inflammatory response, 2) vascular disease, and 3) cancer. Here, two of the five transcripts are highlighted for their immune functions (as the other three had signaling or structural functions). The two with immune functions are: 1) Cytochrome b-p245, beta polypeptide (CYBB) which increased 140% in PRAT after 1 wk of LOCK, and 2) Lipocalin (LCN2) mRNA increased 70% on the 7^th^ day of LOCK. The CYBB gene encodes a protein called cytochrome b-245, beta subunit. This protein is a subunit of a group of proteins that forms the NADPH oxidase complex which plays an essential role in phagocyte reactive oxygen species production [30]. With regards to the LOCK-induced alteration in Lcn2, increases in metabolic stress, proinflammatory cytokines (TNFα, IL-1β, or IL6), and the intake of certain nutrients (palmitate or oleate) have been shown to increase the expression of this gene [31]. During the inflammatory response, Lcn2 is also increasingly secreted by adipocytes [31]. Intriguingly, our IPA generated networks also identified several major histocompatibility complex transcripts (MHCII, HLA-DMB, and HLA-DMA) upregulated with LOCK that are associated with immune response, supporting that macrophage infiltration into adipose tissue may be occurring after 1 wk of LOCK. Cho et al. [32] noted that when MHCII activity is lost, the detrimental effects of obesity and inflammation are also lost. Therefore, our data again demonstrates that the cessation of routine physical activity leads to a rapid expansion of visceral adipose tissue which is accompanied by increases in inflammatory markers.

Analysis of exclusively down-regulated transcripts produced a top IPA networks with functions including “metabolic disease,” “lipid metabolism,” and “molecular transport.” Several of the down-regulated transcripts in this network have been previously associated with obesity-related metabolic changes. For instance, expression of insulin induced gene 1 (INSIG1) was decreased 1.7-fold in 7 days of LOCK, and was present in the top scoring IPA network inclusive of both up- and down-regulated transcripts. INSIG1 mRNA down-regulation has been observed in adipose tissue from obese mice and humans, and may function to ameliorate adipocyte insulin resistance [33]. Additionally, the down-regulation of FABP4 and CD36 transcripts with LOCK suggests that changes in PRAT following LOCK may mirror changes observed in obesity given findings that obese men display a down-regulated adipose tissue fatty acid trafficking transcriptomic signature compared to lean men [34]. These mRNA expression changes in LOCK are indicative of decreased fatty acid metabolism and increased fatty acid storage, which are in agreement with functional measurements from our previous work which show that triacylglycerol synthesis [1, 35] and mitochondrial glycerol-3-phosphate acyltransferase-1 activity [35] were increased with LOCK. Another transcript downregulated was adiponectin receptor 2 (ADIPOR2). It is known that low levels of plasma adiponectin in obesity cause insulin resistance and type 2 diabetes [36], so we speculate that stopping voluntary running could contribute to adipose tissue insulin resistance. Future studies should perform metabolic profiling following 1 wk of LOCK to determine its influence on adipose tissue insulin resistance.

Assessment of the top 20 down-regulated transcripts at LOCK day 7 showed a down-regulation of the homeobox transcription factors HOXA9 and HOXC10. Fat loss by bariatric surgery produces an up-regulation in homeobox transcripts, including HOXA9, as well as a down-regulation of inflammatory and immune cell function transcripts [37]. Additionally, microarray data from obese children at the time of elective surgery showed expression of HOXC10 mRNA was nearly undetectable in visceral depots, but was detectable in subcutaneous adipose tissue of the same subjects. It is also noteworthy to mention that low HOXC10 is associated with Gene Ontology pathways related to inflammatory and immune responses [38]. Based on the findings that subjects with obesity exhibit low-grade inflammation due to a diminished expression of homeobox transcripts [37], we propose that homeobox transcription factors may lessen inflammation, or may be secondary to changes in adipose tissue mass.

To further assess transcriptional differences between RUN and LOCK, we analyzed transcripts uniquely expressed in either RUN or LOCK. Intriguingly, we observed 244 transcripts unique to LOCK, while only 32 transcripts were expressed exclusively in RUN, suggesting that the cessation of physical activity is accompanied by an increased number of unique genes needed to produce/maintain the effects of LOCK. Analysis of the 244 transcripts exclusively expressed in LOCK revealed increases in mRNAs affecting cell cycle, DNA replication, and cellular assembly and repair, as predicted by IPA network analysis. Of the 10 most highly expressed transcripts exclusive to LOCK, regulation of the cell cycle, type 2 diabetes, adipogenesis and cell proliferation, and inflammation have been associated with topoisomerase II alpha (TOP2A) [39], cyclin-dependent kinase inhibitor 3 (CDKN3) [40], monoclonal antibody Ki-67 (MK167) [41], TPX2, microtubule-associated, homolog (TPX2) [42], cyclin A2 (CCNA2) [43], aurora kinase B (AURKB) [44], and kinesin family member 23 (KIF23) [44]. Taken together with our data demonstrating increased expression of inflammatory networks and probable macrophage activation following LOCK, as well as previous findings demonstrating activated macrophages induce expression of cell cycle proteins such as the cyclins CCNA2 [45] and Cyclin D2 [46], we believe the presence of cell cycle and DNA replication transcripts in LOCK, but not RUN, may be the result of increased numbers of activated macrophages in adipose tissue. Additionally, these findings corroborate our previous observations that cell cycle genes increase following 1 week of LOCK, further suggesting drastic increases in cell cycle progression of adipose tissue follow the cessation of physical activity [4].

Limitations of the study follow. Because rats in RUN were given free access to voluntary running wheels, the time between the last exercise bout and sacrifice was variable between rats (on average between 4–6 hours), potentially influencing the rapidly changing transcriptomic profile. Due to economic constraints, we could only select one adipose tissue depot for transcriptomics. We selected the perirenal adipose tissue in our analysis for the following reasons: 1) its relatively large size in the intra-abdominal cavity; 2) its mass increased 78% in 7 days of LOCK; 3) the mass of PRAT was 6X and 7X greater than the OMAT in RUN and LOCK, respectively; and 4) omental adipose tissue has pancreatic transcripts due to the rats pancreas not being one discrete tissue. Additionally, the observed alterations may be resultant to, rather than a facilitator of, the rapid increase adipose tissue size in LOCK. Future studies must determine which transcriptomic alterations are resultant to the cessation of physical activity and facilitated weight gain and which are the result of increases in adiposity. Finally and importantly, the unexpected increase in transcripts whose gene functions are unhealthy suggests the average increase of 78% in perirenal adipose tissue mass over the 7-day LOCK period could have detrimental function.

### Conclusions and Perspectives for Future Research

Our data suggests that 1 week of LOCK is capable of producing transcriptomic changes comparable to those observed after several months of high-fat feeding. Moreover, there is rapid adipose tissue growth, and this growth is associated with increases in immune/inflammatory markers. We postulate that two important themes can be gleaned from these data:

1. The 7-day LOCK model transiently decreases the caloric value of physical activity more than the decrease in food intake, producing a temporary positive caloric balance that increases visceral adipose tissue. In diet-induced obesity models, however, high-fat feeding is imposed upon rodents that maintain sedentary physical activity levels. In other words, high-fat feeding is imposed without altering physical activity to produce a greater positive caloric balance. Our data suggest that this is an important methodological consideration when studying the etiology of obesity because the rate of adipose tissue accretion and the presentation of a pro-inflammatory adipose tissue phenotype is drastically different between these two models.
2. Based upon our current data, along with data from our other LOCK studies, we postulate that the catch-up enlargement of adipose tissue upon the cessation in routine physical activity may be a natural survival reflex/adaptation that was inherited from mammalian pre-historic ancestors. Alternatively stated, animals that were able to quickly accrue visceral adipose tissue in times of ‘feast and famine’ may have had the advantage of harnessing sufficient caloric stores during prolonged states of deprived energy intake; this being related to Neel’s thrifty gene hypothesis [47]. However, in the LOCK model, where there is continuous *ad libitum* feeding, this ancestrally-inherited visceral adipose tissue trait is mal-adapted and potentially leads to a rapid development in adipose tissue-initiated low-grade inflammation. Therefore, this study underscores the importance of maintaining routine physical activity levels in our current-day ‘*ad libitum* society’ in order to sustain a relatively healthy adipose tissue phenotype.

Finally, we contend that more research is needed with the LOCK model in order to determine how other tissues (i.e., brain, endocrine glands, etc.) respond to the cessation of routine physical activity. Additionally, future studies should analyze the effects of exercise cessation on other visceral fat depots, as well as transcriptomic differences in subcutaneous vs. visceral deports in response to LOCK.

## Supporting Information

S1 Table Complete list of up- and down-regulated genes differentially expressed between RUN and LOCK, sorted by magnitude of fold change. (DOCX)

S2 Table Complete list of transcripts expressed only in RUN, sorted by magnitude of RPKM. (DOCX)

S3 Table Complete list of transcripts expressed only in LOCK, sorted by magnitude of RPKM. (DOCX)
