# Supplementary material for: Rapid Alterations in Perirenal Adipose Tissue Transcriptomic Networks with Cessation of Voluntary Running
Source: PLoS One. 2015 Dec 17;10(12):e0145229. doi: 10.1371/journal.pone.0145229 (PMC4683046; doi:10.1371/journal.pone.0145229)
Supplement: S1 Table — (DOCX) [file pone.0145229.s001.docx]

S1 Table: Complete list of up- and down-regulated genes differentially expressed between RUN and LOCK, sorted by magnitude of fold change.

| **Transcript Description** | **RUN Average RPKM** | **LOCK Average RPKM** | **Fold-change (LOCK/RUN)** | **p-value** |
| --- | --- | --- | --- | --- |
| *Up-regulated in LOCK* |  |  |  |  |
| Retnlg | 3.93 | 29.08 | 7.40 | 0.049 |
| Clec11A | 2.09 | 11.52 | 5.52 | 0.046 |
| Lox | 8.57 | 38.97 | 4.55 | 0.034 |
| Sfrp1 | 4.22 | 18.08 | 4.28 | 0.040 |
| Ptgis | 8.75 | 35.55 | 4.07 | 0.034 |
| Flrt2 | 2.78 | 11.08 | 3.99 | 0.032 |
| Ccl13 | 9.93 | 36.05 | 3.63 | 0.047 |
| Mt2A | 3.77 | 13.16 | 3.49 | 0.021 |
| Lum | 42.35 | 146.95 | 3.47 | 0.045 |
| C3 | 3.26 | 11.17 | 3.43 | 0.048 |
| Fn1 | 59.75 | 202.91 | 3.40 | 0.044 |
| Kiaa0101 | 2.80 | 9.47 | 3.38 | 0.007 |
| Spon1 | 7.99 | 26.80 | 3.35 | 0.029 |
| Eln | 35.10 | 115.71 | 3.30 | 0.011 |
| Lyve1 | 20.74 | 68.15 | 3.29 | 0.029 |
| Mfap4 | 16.30 | 53.47 | 3.28 | 0.002 |
| Dpysl3 | 4.18 | 13.44 | 3.22 | 0.014 |
| Fhl2 | 2.74 | 8.69 | 3.17 | 0.039 |
| Dpysl3 | 5.30 | 16.73 | 3.15 | 0.015 |
| Col12A1 | 2.98 | 9.29 | 3.12 | 0.003 |
| S100A4 | 45.32 | 139.75 | 3.08 | 0.028 |
| Serpine2 | 9.57 | 28.22 | 2.95 | 0.041 |
| Cd44 | 5.59 | 16.46 | 2.94 | 0.037 |
| Msr1 | 6.25 | 18.36 | 2.94 | 0.041 |
| Hmgb2 | 13.10 | 38.30 | 2.92 | 0.019 |
| Slc43A2 | 3.30 | 9.31 | 2.83 | 0.035 |
| Itgam | 6.83 | 19.10 | 2.80 | 0.042 |
| Lmnb1 | 3.27 | 9.03 | 2.77 | 0.005 |
| Col3A1 | 544.15 | 1490.37 | 2.74 | 0.019 |
| Col1A1 | 143.17 | 391.27 | 2.73 | 0.008 |
| Col1A2 | 137.44 | 373.67 | 2.72 | 0.006 |
| Cthrc1 | 2.20 | 5.91 | 2.68 | 0.001 |
| Lyz | 119.87 | 319.50 | 2.67 | 0.046 |
| Ccdc85C | 2.61 | 6.95 | 2.66 | 0.041 |
| Cybb | 9.27 | 24.31 | 2.62 | 0.026 |
| Mcm6 | 2.54 | 6.63 | 2.61 | 0.007 |
| Glt8D2 | 3.48 | 8.94 | 2.57 | 0.038 |
| Cd68 | 14.91 | 37.80 | 2.53 | 0.029 |
| Mcm2 | 2.84 | 7.15 | 2.52 | 0.009 |
| Ltbp4 | 23.69 | 59.61 | 2.52 | 0.048 |
| Mgp | 96.44 | 242.64 | 2.52 | 0.022 |
| Lilra6 | 2.30 | 5.80 | 2.51 | 0.038 |
| Cd163 | 15.98 | 40.09 | 2.51 | 0.040 |
| Fgf18 | 2.50 | 6.26 | 2.51 | 0.036 |
| Myh10 | 2.04 | 5.08 | 2.49 | 0.008 |
| Soat1 | 2.71 | 6.69 | 2.47 | 0.020 |
| Timp1 | 14.26 | 34.99 | 2.45 | 0.015 |
| Tubb6 | 11.88 | 29.14 | 2.45 | 0.008 |
| Tk1 | 3.67 | 8.96 | 2.44 | 0.003 |
| Bmp6 | 2.89 | 7.05 | 2.44 | 0.016 |
| Nfam1 | 3.79 | 9.23 | 2.44 | 0.030 |
| Smoc2 | 6.69 | 16.27 | 2.43 | 0.004 |
| Thy1 | 9.70 | 23.52 | 2.42 | 0.005 |
| Col16A1 | 6.16 | 14.89 | 2.42 | 0.015 |
| Pstpip1 | 2.12 | 5.11 | 2.41 | 0.016 |
| Ccr2 | 4.61 | 11.13 | 2.41 | 0.012 |
| Samsn1 | 2.99 | 7.09 | 2.37 | 0.046 |
| Cybb | 4.33 | 10.22 | 2.36 | 0.040 |
| Irf8 | 5.59 | 13.12 | 2.35 | 0.006 |
| Inpp5F | 10.12 | 23.78 | 2.35 | 0.032 |
| C1Qtnf6 | 10.42 | 24.46 | 2.35 | 0.026 |
| Myof | 6.91 | 16.21 | 2.35 | 0.038 |
| Tifab | 2.02 | 4.72 | 2.34 | 0.014 |
| Glis2 | 6.25 | 14.64 | 2.34 | 0.042 |
| Haus4 | 2.04 | 4.75 | 2.33 | 0.014 |
| Bmp1 | 11.95 | 27.63 | 2.31 | 0.029 |
| Fstl1 | 57.42 | 132.42 | 2.31 | 0.000 |
| Bok | 2.44 | 5.61 | 2.30 | 0.028 |
| Cmahp | 6.26 | 14.34 | 2.29 | 0.042 |
| H1Fx | 3.22 | 7.37 | 2.29 | 0.046 |
| Cd34 | 50.34 | 115.07 | 2.29 | 0.017 |
| Col6A3 | 11.07 | 25.26 | 2.28 | 0.005 |
| Pdk3 | 2.25 | 5.12 | 2.28 | 0.044 |
| C18Orf54 | 4.61 | 10.51 | 2.28 | 0.043 |
| Cd64 | 8.16 | 18.59 | 2.28 | 0.048 |
| Bicc1 | 4.49 | 10.21 | 2.27 | 0.021 |
| Pi15 | 2.21 | 5.00 | 2.26 | 0.026 |
| Ptpn7 | 2.18 | 4.90 | 2.25 | 0.012 |
| Tcf19 | 4.15 | 9.33 | 2.25 | 0.005 |
| Col14A1 | 23.17 | 51.99 | 2.24 | 0.006 |
| Mrc2 | 6.10 | 13.60 | 2.23 | 0.026 |
| Thbs3 | 3.45 | 7.68 | 2.23 | 0.003 |
| Apitd1 | 2.06 | 4.57 | 2.22 | 0.011 |
| Loxl1 | 16.56 | 36.70 | 2.22 | 0.003 |
| Ndn | 3.50 | 7.75 | 2.21 | 0.028 |
| Sla | 3.24 | 7.12 | 2.20 | 0.032 |
| Siae | 3.65 | 8.00 | 2.19 | 0.048 |
| Wfdc1 | 6.22 | 13.61 | 2.19 | 0.010 |
| Arhgap30 | 3.61 | 7.85 | 2.18 | 0.012 |
| Rab32 | 5.39 | 11.69 | 2.17 | 0.040 |
| Fkbp10 | 7.41 | 16.05 | 2.17 | 0.026 |
| Rcn3 | 20.82 | 45.11 | 2.17 | 0.031 |
| Atad2 | 2.80 | 6.02 | 2.15 | 0.005 |
| Vgll3 | 6.65 | 14.26 | 2.15 | 0.001 |
| Clec4A | 14.48 | 30.93 | 2.14 | 0.020 |
| Ms4A6A | 3.96 | 8.44 | 2.13 | 0.041 |
| Gpr133 | 5.00 | 10.66 | 2.13 | 0.009 |
| Atp8B2 | 6.50 | 13.85 | 2.13 | 0.018 |
| Dmd | 2.69 | 5.73 | 2.13 | 0.025 |
| C3Orf70 | 4.58 | 9.71 | 2.12 | 0.050 |
| Arhgap11A | 2.93 | 6.20 | 2.12 | 0.001 |
| Cercam | 6.45 | 13.66 | 2.12 | 0.013 |
| Ccr5 | 4.52 | 9.53 | 2.11 | 0.012 |
| Zmynd15 | 3.68 | 7.76 | 2.11 | 0.048 |
| B4Galt5 | 6.41 | 13.51 | 2.11 | 0.022 |
| Napsb | 4.43 | 9.31 | 2.10 | 0.045 |
| Gpm6B | 2.20 | 4.62 | 2.10 | 0.014 |
| Tlr2 | 3.50 | 7.32 | 2.09 | 0.029 |
| Ril | 7.00 | 14.58 | 2.08 | 0.037 |
| Nek6 | 6.37 | 13.26 | 2.08 | 0.006 |
| Adcy7 | 2.17 | 4.52 | 2.08 | 0.006 |
| Col6A5 | 2.98 | 6.20 | 2.08 | 0.001 |
| Antxr1 | 9.28 | 19.28 | 2.08 | 0.043 |
| Stac | 3.60 | 7.46 | 2.07 | 0.015 |
| Cfb | 20.13 | 41.65 | 2.07 | 0.048 |
| Hck | 5.21 | 10.79 | 2.07 | 0.013 |
| Zmym6Nb | 3.28 | 6.76 | 2.06 | 0.035 |
| Pla2g4a | 4.14 | 8.52 | 2.06 | 0.005 |
| Erbb3 | 2.58 | 5.30 | 2.05 | 0.002 |
| Myo1F | 5.02 | 10.30 | 2.05 | 0.017 |
| B3galnt1 | 5.91 | 12.12 | 2.05 | 0.037 |
| Ttc7A | 3.05 | 6.25 | 2.05 | 0.030 |
| Tifa | 3.93 | 8.05 | 2.05 | 0.038 |
| Pla1A | 4.03 | 8.24 | 2.05 | 0.022 |
| Pcolce | 40.45 | 82.66 | 2.04 | 0.022 |
| Wee1 | 3.89 | 7.94 | 2.04 | 0.046 |
| Fbn1 | 73.21 | 149.10 | 2.04 | 0.001 |
| Calhm2 | 4.47 | 9.10 | 2.04 | 0.009 |
| Dnm1 | 3.76 | 7.63 | 2.03 | 0.007 |
| Folr2 | 25.20 | 51.06 | 2.03 | 0.033 |
| Ptplad2 | 5.93 | 12.02 | 2.03 | 0.035 |
| Gja1 | 22.67 | 45.88 | 2.02 | 0.042 |
| Cotl1 | 25.81 | 52.22 | 2.02 | 0.017 |
| Hmox1 | 14.08 | 28.38 | 2.02 | 0.008 |
| Trim47 | 5.26 | 10.55 | 2.01 | 0.046 |
| Cyth4 | 7.64 | 15.33 | 2.01 | 0.020 |
| Jdp2 | 6.49 | 13.01 | 2.01 | 0.015 |
| Col4A4 | 2.72 | 5.42 | 1.99 | 0.022 |
| Kctd17 | 3.53 | 7.03 | 1.99 | 0.030 |
| Pmf1 | 4.10 | 8.15 | 1.99 | 0.006 |
| Tmpo | 8.98 | 17.86 | 1.99 | 0.040 |
| Aldh18A1 | 2.84 | 5.65 | 1.99 | 0.008 |
| Mcm4 | 3.83 | 7.61 | 1.99 | 0.002 |
| Nckap1L | 4.45 | 8.81 | 1.98 | 0.023 |
| Slit3 | 15.81 | 31.30 | 1.98 | 0.013 |
| Emp3 | 30.95 | 61.22 | 1.98 | 0.030 |
| C1Qc | 105.18 | 207.91 | 1.98 | 0.048 |
| Ikzf1 | 2.07 | 4.08 | 1.98 | 0.034 |
| Lgals3Bp | 42.04 | 82.65 | 1.97 | 0.043 |
| Cebpd | 5.28 | 10.38 | 1.96 | 0.033 |
| Vnn1 | 5.36 | 10.53 | 1.96 | 0.017 |
| Lgi4 | 2.11 | 4.11 | 1.95 | 0.041 |
| Adamts2 | 12.90 | 25.12 | 1.95 | 0.008 |
| Ccrl1 | 12.77 | 24.84 | 1.95 | 0.016 |
| Itgb2 | 10.09 | 19.61 | 1.94 | 0.031 |
| Rab7B | 5.38 | 10.46 | 1.94 | 0.035 |
| F13A1 | 38.29 | 74.21 | 1.94 | 0.024 |
| Prkcb | 2.07 | 4.02 | 1.94 | 0.024 |
| Bcat1 | 2.82 | 5.46 | 1.94 | 0.004 |
| Fgd2 | 2.41 | 4.66 | 1.94 | 0.044 |
| Khdrbs3 | 4.99 | 9.66 | 1.94 | 0.010 |
| Fermt3 | 5.47 | 10.57 | 1.93 | 0.026 |
| Ncf4 | 4.86 | 9.38 | 1.93 | 0.013 |
| Gna15 | 2.15 | 4.15 | 1.93 | 0.023 |
| Lama2 | 9.92 | 19.10 | 1.93 | 0.019 |
| B4Galt5 | 8.65 | 16.63 | 1.92 | 0.021 |
| Tubb | 71.69 | 137.77 | 1.92 | 0.015 |
| Dlg4 | 2.49 | 4.79 | 1.92 | 0.045 |
| Fxyd5 | 13.71 | 26.34 | 1.92 | 0.021 |
| Dctpp1 | 2.71 | 5.19 | 1.92 | 0.007 |
| Hla-Dmb | 18.20 | 34.83 | 1.91 | 0.031 |
| Kdelr3 | 12.83 | 24.54 | 1.91 | 0.010 |
| Numbl | 4.61 | 8.82 | 1.91 | 0.030 |
| Bmp7 | 3.07 | 5.86 | 1.91 | 0.035 |
| Ptprc | 7.47 | 14.25 | 1.91 | 0.027 |
| Plxnb2 | 14.11 | 26.89 | 1.91 | 0.033 |
| Elf4 | 3.07 | 5.85 | 1.91 | 0.009 |
| Was | 3.05 | 5.81 | 1.91 | 0.041 |
| Il4R | 13.51 | 25.69 | 1.90 | 0.010 |
| Cp | 11.42 | 21.63 | 1.89 | 0.005 |
| Cyba | 21.94 | 41.56 | 1.89 | 0.032 |
| Plagl1 | 9.50 | 17.99 | 1.89 | 0.042 |
| Grb10 | 12.29 | 23.26 | 1.89 | 0.027 |
| Tnxb | 23.74 | 44.94 | 1.89 | 0.023 |
| Selplg | 6.01 | 11.37 | 1.89 | 0.019 |
| Tram2 | 2.84 | 5.37 | 1.89 | 0.008 |
| Syk | 3.54 | 6.67 | 1.89 | 0.018 |
| Pik3Cd | 3.59 | 6.76 | 1.89 | 0.032 |
| Scarf2 | 2.38 | 4.48 | 1.88 | 0.040 |
| C5Ar1 | 6.66 | 12.54 | 1.88 | 0.017 |
| Nbl1 | 55.24 | 103.95 | 1.88 | 0.041 |
| Hla-Dma | 11.30 | 21.23 | 1.88 | 0.002 |
| Arl11 | 6.23 | 11.70 | 1.88 | 0.021 |
| Bst1 | 3.27 | 6.13 | 1.87 | 0.041 |
| Tyrobp | 37.87 | 70.92 | 1.87 | 0.038 |
| Frmd4B | 3.57 | 6.67 | 1.87 | 0.032 |
| Btk | 2.88 | 5.37 | 1.87 | 0.044 |
| Fxyd2 | 36.10 | 67.34 | 1.87 | 0.027 |
| Kdelc2 | 17.57 | 32.68 | 1.86 | 0.032 |
| Fxyd3 | 6.41 | 11.90 | 1.86 | 0.037 |
| Tns3 | 10.03 | 18.62 | 1.86 | 0.024 |
| Sfxn3 | 10.37 | 19.24 | 1.86 | 0.049 |
| Il13Ra1 | 9.88 | 18.30 | 1.85 | 0.041 |
| Me2 | 6.44 | 11.92 | 1.85 | 0.049 |
| Oas2 | 2.96 | 5.48 | 1.85 | 0.029 |
| Armcx2 | 2.39 | 4.43 | 1.85 | 0.049 |
| Pcolce | 3.31 | 6.12 | 1.85 | 0.043 |
| Alox5 | 6.35 | 11.75 | 1.85 | 0.011 |
| Csf1R | 39.79 | 73.54 | 1.85 | 0.041 |
| Vcan | 2.43 | 4.49 | 1.85 | 0.049 |
| H2Afx | 7.62 | 14.09 | 1.85 | 0.011 |
| Tns3 | 3.18 | 5.87 | 1.85 | 0.001 |
| Src | 3.59 | 6.62 | 1.85 | 0.026 |
| Bmp4 | 5.47 | 10.09 | 1.85 | 0.033 |
| Pdlim1 | 19.05 | 35.14 | 1.84 | 0.011 |
| Il34 | 11.06 | 20.40 | 1.84 | 0.040 |
| Capg | 21.36 | 39.39 | 1.84 | 0.013 |
| Htra1 | 79.03 | 145.72 | 1.84 | 0.004 |
| Emp1 | 67.58 | 124.54 | 1.84 | 0.016 |
| Gng2 | 4.16 | 7.67 | 1.84 | 0.045 |
| Gpm6B | 2.22 | 4.08 | 1.84 | 0.047 |
| Myo1G | 3.12 | 5.73 | 1.84 | 0.015 |
| Lyl1 | 3.77 | 6.92 | 1.84 | 0.028 |
| Igsf8 | 3.91 | 7.18 | 1.84 | 0.013 |
| Rasgrp4 | 2.88 | 5.28 | 1.83 | 0.007 |
| Camkk1 | 3.47 | 6.36 | 1.83 | 0.017 |
| Cp | 5.48 | 9.99 | 1.82 | 0.004 |
| Mrgprf | 3.88 | 7.07 | 1.82 | 0.010 |
| Hcls1 | 6.47 | 11.78 | 1.82 | 0.022 |
| Bcl2A1 | 9.61 | 17.49 | 1.82 | 0.043 |
| Tmem106A | 4.09 | 7.42 | 1.82 | 0.022 |
| A4 | 7.81 | 14.18 | 1.82 | 0.032 |
| Snx10 | 3.25 | 5.90 | 1.81 | 0.028 |
| Dab2 | 28.84 | 52.17 | 1.81 | 0.050 |
| Kirrel | 7.47 | 13.51 | 1.81 | 0.000 |
| Aspn | 20.55 | 37.14 | 1.81 | 0.003 |
| Pld4 | 12.26 | 22.14 | 1.81 | 0.026 |
| Znf367 | 2.26 | 4.08 | 1.81 | 0.002 |
| Itga5 | 7.91 | 14.26 | 1.80 | 0.026 |
| Ptpro | 2.58 | 4.66 | 1.80 | 0.014 |
| Lcp1 | 23.09 | 41.59 | 1.80 | 0.012 |
| Enpp3 | 17.54 | 31.57 | 1.80 | 0.028 |
| Calml4 | 4.42 | 7.95 | 1.80 | 0.019 |
| Wnt2 | 2.08 | 3.73 | 1.80 | 0.031 |
| Akt3 | 4.67 | 8.38 | 1.80 | 0.032 |
| Ly86 | 4.69 | 8.43 | 1.80 | 0.045 |
| S1Pr2 | 5.20 | 9.34 | 1.80 | 0.020 |
| Epha1 | 9.28 | 16.65 | 1.79 | 0.027 |
| Tbc1D2B | 12.85 | 23.06 | 1.79 | 0.050 |
| Galnt10 | 2.72 | 4.88 | 1.79 | 0.035 |
| Alox5Ap | 19.10 | 34.24 | 1.79 | 0.028 |
| Cfb | 103.23 | 184.87 | 1.79 | 0.047 |
| Csf2Ra | 11.50 | 20.59 | 1.79 | 0.038 |
| Mrc1 | 21.60 | 38.67 | 1.79 | 0.046 |
| Efemp2 | 9.89 | 17.67 | 1.79 | 0.014 |
| Ednra | 2.48 | 4.44 | 1.79 | 0.009 |
| Rps6Ka1 | 3.33 | 5.93 | 1.78 | 0.050 |
| Hmha1 | 4.36 | 7.78 | 1.78 | 0.034 |
| Tmem37 | 3.25 | 5.79 | 1.78 | 0.039 |
| Plec | 5.28 | 9.41 | 1.78 | 0.016 |
| Coro1A | 15.78 | 28.12 | 1.78 | 0.020 |
| Gnb4 | 5.39 | 9.58 | 1.78 | 0.019 |
| Efemp2 | 3.38 | 6.02 | 1.78 | 0.007 |
| Ezh2 | 2.66 | 4.72 | 1.78 | 0.023 |
| Ubtd2 | 5.52 | 9.79 | 1.77 | 0.024 |
| Tmem98 | 18.49 | 32.75 | 1.77 | 0.000 |
| Gpr124 | 6.07 | 10.74 | 1.77 | 0.029 |
| Fbln2 | 31.04 | 54.98 | 1.77 | 0.002 |
| Gdi | 43.45 | 76.94 | 1.77 | 0.048 |
| Mfrp | 4.00 | 7.08 | 1.77 | 0.016 |
| Cdsn | 4.41 | 7.81 | 1.77 | 0.025 |
| Celf2 | 3.22 | 5.69 | 1.77 | 0.025 |
| Rt1-S3 | 48.82 | 86.20 | 1.77 | 0.030 |
| Mcm7 | 6.24 | 11.02 | 1.77 | 0.027 |
| Ccnd1 | 10.73 | 18.94 | 1.76 | 0.024 |
| Ppic | 22.42 | 39.55 | 1.76 | 0.013 |
| Slfn8 | 8.90 | 15.69 | 1.76 | 0.036 |
| Bin2 | 3.49 | 6.16 | 1.76 | 0.031 |
| Crip | 97.22 | 171.05 | 1.76 | 0.025 |
| Neurl1b | 4.69 | 8.24 | 1.76 | 0.008 |
| Ang | 3.04 | 5.33 | 1.75 | 0.035 |
| Rnasel | 3.91 | 6.86 | 1.75 | 0.045 |
| Pcdh18 | 2.95 | 5.16 | 1.75 | 0.007 |
| Cilp | 6.88 | 12.01 | 1.75 | 0.001 |
| Aif1 | 9.73 | 16.97 | 1.74 | 0.050 |
| Itga4 | 2.51 | 4.37 | 1.74 | 0.006 |
| Inpp5D | 2.60 | 4.52 | 1.74 | 0.034 |
| Ggt5 | 6.82 | 11.87 | 1.74 | 0.046 |
| Pcsk6 | 15.26 | 26.56 | 1.74 | 0.000 |
| Rt1-T24-3 | 18.64 | 32.44 | 1.74 | 0.001 |
| Dmbt1 | 6.62 | 11.51 | 1.74 | 0.035 |
| Loxl3 | 2.66 | 4.63 | 1.74 | 0.028 |
| Il17Ra | 7.86 | 13.67 | 1.74 | 0.015 |
| Ifi44 | 16.71 | 29.05 | 1.74 | 0.044 |
| Cmtm3 | 9.05 | 15.70 | 1.74 | 0.042 |
| Il6R | 8.18 | 14.17 | 1.73 | 0.024 |
| Nucb2 | 29.86 | 51.70 | 1.73 | 0.020 |
| Ralgds/Af-6 | 6.22 | 10.77 | 1.73 | 0.030 |
| Ptprj | 4.94 | 8.55 | 1.73 | 0.020 |
| Matn2 | 8.07 | 13.96 | 1.73 | 0.000 |
| Pkdcc | 21.06 | 36.38 | 1.73 | 0.018 |
| Ugt1A7 | 2.65 | 4.57 | 1.72 | 0.000 |
| Pycard | 16.19 | 27.87 | 1.72 | 0.028 |
| Rassf5 | 2.38 | 4.09 | 1.72 | 0.038 |
| Arpc1B | 95.48 | 164.22 | 1.72 | 0.001 |
| Med13L | 4.55 | 7.82 | 1.72 | 0.045 |
| Plekho2 | 9.28 | 15.93 | 1.72 | 0.008 |
| Samd1 | 10.34 | 17.72 | 1.71 | 0.032 |
| Sffv | 10.80 | 18.51 | 1.71 | 0.008 |
| Slc37a2 | 2.17 | 3.72 | 1.71 | 0.013 |
| Rbpj | 5.62 | 9.61 | 1.71 | 0.026 |
| Leprel2 | 16.17 | 27.60 | 1.71 | 0.021 |
| Schmidt-Ruppin A-2 | 4.03 | 6.89 | 1.71 | 0.041 |
| Sld5 Homolog | 2.63 | 4.48 | 1.71 | 0.024 |
| Kiaa0513 | 4.59 | 7.83 | 1.71 | 0.035 |
| Maged2 | 14.05 | 23.92 | 1.70 | 0.041 |
| Gef | 4.18 | 7.11 | 1.70 | 0.050 |
| C2Orf40 | 2.77 | 4.71 | 1.70 | 0.019 |
| Ig | 52.68 | 89.53 | 1.70 | 0.001 |
| Cchcr1 | 2.86 | 4.86 | 1.70 | 0.046 |
| Myadm | 40.60 | 68.88 | 1.70 | 0.038 |
| Activator 1 | 3.94 | 6.67 | 1.70 | 0.000 |
| Dlgap5 | 3.06 | 5.19 | 1.70 | 0.010 |
| Snai1 | 4.13 | 7.00 | 1.70 | 0.015 |
| Dapp1 | 2.24 | 3.79 | 1.69 | 0.026 |
| Entpd1 | 12.19 | 20.66 | 1.69 | 0.038 |
| Fscn1 | 17.56 | 29.71 | 1.69 | 0.027 |
| E2f1 | 3.05 | 5.16 | 1.69 | 0.000 |
| Fam20C | 3.70 | 6.25 | 1.69 | 0.048 |
| Dusp18 | 3.38 | 5.71 | 1.69 | 0.011 |
| Lcn2 | 5.37 | 9.08 | 1.69 | 0.005 |
| Atp2B1 | 9.57 | 16.16 | 1.69 | 0.026 |
| Ten1 | 2.67 | 4.51 | 1.69 | 0.008 |
| Dck | 2.12 | 3.58 | 1.69 | 0.006 |
| Rgs18 | 7.56 | 12.75 | 1.69 | 0.047 |
| Mast4 | 2.56 | 4.32 | 1.69 | 0.019 |
| Msn | 21.78 | 36.72 | 1.69 | 0.018 |
| Bcl3 | 3.18 | 5.35 | 1.68 | 0.030 |
| Smc2 | 4.26 | 7.15 | 1.68 | 0.003 |
| Fam149A | 3.90 | 6.53 | 1.68 | 0.007 |
| Sash3 | 2.96 | 4.95 | 1.67 | 0.021 |
| Pdia4 | 32.66 | 54.68 | 1.67 | 0.028 |
| Slco2A1 | 4.08 | 6.82 | 1.67 | 0.019 |
| Fyn | 5.91 | 9.88 | 1.67 | 0.049 |
| Igdcc4 | 3.60 | 6.00 | 1.67 | 0.020 |
| Ikbip | 13.95 | 23.29 | 1.67 | 0.026 |
| Olfml2B | 6.13 | 10.23 | 1.67 | 0.034 |
| Rgd1563520 | 2.76 | 4.60 | 1.67 | 0.005 |
| Pik3ap1 | 2.92 | 4.87 | 1.67 | 0.034 |
| Rcan3 | 2.86 | 4.76 | 1.67 | 0.002 |
| Ndk1 | 11.96 | 19.90 | 1.66 | 0.013 |
| Rbpj | 12.46 | 20.73 | 1.66 | 0.030 |
| Clic1 | 55.04 | 91.48 | 1.66 | 0.031 |
| Prkcd | 7.10 | 11.79 | 1.66 | 0.020 |
| Cd86 | 2.30 | 3.82 | 1.66 | 0.024 |
| C15Orf48 | 2.81 | 4.66 | 1.66 | 0.026 |
| Cnn2 | 13.14 | 21.80 | 1.66 | 0.014 |
| Nasp | 5.31 | 8.81 | 1.66 | 0.029 |
| Tpm4 | 121.53 | 201.48 | 1.66 | 0.003 |
| Nrm | 2.80 | 4.63 | 1.66 | 0.042 |
| Tgfb1 | 12.18 | 20.17 | 1.66 | 0.039 |
| Ap1S2 | 9.04 | 14.96 | 1.66 | 0.031 |
| Calm3 | 116.76 | 193.14 | 1.65 | 0.043 |
| Tgfb1I1 | 7.22 | 11.94 | 1.65 | 0.038 |
| Loc308990 | 2.96 | 4.90 | 1.65 | 0.033 |
| Chn2 | 2.04 | 3.37 | 1.65 | 0.010 |
| Axl | 25.18 | 41.56 | 1.65 | 0.007 |
| Orai2 | 2.34 | 3.86 | 1.65 | 0.024 |
| Timp2 | 131.46 | 216.81 | 1.65 | 0.036 |
| Heph | 8.12 | 13.39 | 1.65 | 0.022 |
| Tmem246 | 2.06 | 3.39 | 1.65 | 0.003 |
| Rt1-M3-1 | 6.00 | 9.87 | 1.64 | 0.048 |
| Arap1 | 9.02 | 14.80 | 1.64 | 0.022 |
| Qser1 | 2.76 | 4.52 | 1.64 | 0.012 |
| Tnks | 3.28 | 5.39 | 1.64 | 0.017 |
| Cdk5Rap2 | 2.07 | 3.40 | 1.64 | 0.006 |
| Ctsk | 19.44 | 31.85 | 1.64 | 0.003 |
| Gxylt2 | 3.22 | 5.28 | 1.64 | 0.023 |
| Lsp1 | 27.47 | 44.97 | 1.64 | 0.016 |
| Nid-2 | 21.09 | 34.50 | 1.64 | 0.014 |
| Gli1 | 2.30 | 3.77 | 1.64 | 0.046 |
| Loc100360462 | 2.26 | 3.70 | 1.63 | 0.008 |
| Efhd2 | 17.92 | 29.22 | 1.63 | 0.029 |
| Smc4 | 8.28 | 13.50 | 1.63 | 0.008 |
| Dnmt1 | 4.02 | 6.54 | 1.63 | 0.011 |
| V-Fgr | 3.79 | 6.16 | 1.63 | 0.011 |
| Ecm1 | 31.80 | 51.59 | 1.62 | 0.003 |
| Hcst | 2.46 | 3.99 | 1.62 | 0.037 |
| Gpc6 | 2.67 | 4.32 | 1.62 | 0.014 |
| Tyro3 | 2.12 | 3.42 | 1.62 | 0.042 |
| Sept11 | 6.70 | 10.82 | 1.62 | 0.005 |
| Myh9 | 57.99 | 93.64 | 1.61 | 0.023 |
| Kctd12 | 11.58 | 18.70 | 1.61 | 0.043 |
| Herpesvirus Entry Mediator B | 6.38 | 10.30 | 1.61 | 0.035 |
| Tnfrsf12A | 5.78 | 9.33 | 1.61 | 0.029 |
| Dact3 | 2.01 | 3.24 | 1.61 | 0.049 |
| Map4K1 | 3.40 | 5.49 | 1.61 | 0.012 |
| Mafb | 9.63 | 15.52 | 1.61 | 0.018 |
| Dnm1 | 5.26 | 8.48 | 1.61 | 0.030 |
| Card6 | 5.71 | 9.20 | 1.61 | 0.045 |
| Casp12 | 9.07 | 14.61 | 1.61 | 0.025 |
| Ptpn6 | 10.57 | 17.01 | 1.61 | 0.040 |
| Gas7 | 17.43 | 28.04 | 1.61 | 0.020 |
| Myo5a | 4.31 | 6.94 | 1.61 | 0.003 |
| Cdk6 | 3.93 | 6.32 | 1.61 | 0.004 |
| Wipf1 | 8.88 | 14.27 | 1.61 | 0.029 |
| Loc100364588 | 15.30 | 24.59 | 1.61 | 0.041 |
| Tgfbr3 | 30.66 | 49.22 | 1.60 | 0.042 |
| Ifi30 | 37.06 | 59.45 | 1.60 | 0.039 |
| Ltc4S | 5.67 | 9.09 | 1.60 | 0.009 |
| Plac9 | 29.43 | 47.11 | 1.60 | 0.003 |
| Bmp2K | 9.81 | 15.70 | 1.60 | 0.009 |
| Loc100366216 | 18.20 | 29.10 | 1.60 | 0.045 |
| Trafd1 | 23.79 | 38.03 | 1.60 | 0.050 |
| Flnb | 9.35 | 14.94 | 1.60 | 0.021 |
| Myo1E | 4.39 | 7.00 | 1.60 | 0.029 |
| Soga1 | 2.72 | 4.34 | 1.59 | 0.002 |
| Pxn | 14.62 | 23.30 | 1.59 | 0.036 |
| Amdhd2 | 4.28 | 6.82 | 1.59 | 0.046 |
| Flna | 35.43 | 56.44 | 1.59 | 0.014 |
| Dbn1 | 5.34 | 8.50 | 1.59 | 0.004 |
| Glt8D1 | 11.39 | 18.13 | 1.59 | 0.044 |
| Lig1 | 5.91 | 9.41 | 1.59 | 0.009 |
| Fam3C | 4.55 | 7.24 | 1.59 | 0.035 |
| Shf | 3.12 | 4.96 | 1.59 | 0.031 |
| Loc678762 | 2.34 | 3.71 | 1.59 | 0.045 |
| Col5A2 | 70.82 | 112.01 | 1.58 | 0.007 |
| Fam114A1 | 17.79 | 28.11 | 1.58 | 0.002 |
| Man1C1 | 7.33 | 11.57 | 1.58 | 0.028 |
| Pabpc1 | 14.08 | 22.23 | 1.58 | 0.002 |
| Txndc16 | 2.95 | 4.66 | 1.58 | 0.042 |
| Mfap5 | 63.21 | 99.67 | 1.58 | 0.030 |
| Dpysl2 | 18.47 | 29.09 | 1.58 | 0.034 |
| Cd276 | 12.09 | 19.00 | 1.57 | 0.014 |
| Cysltr1 | 3.74 | 5.88 | 1.57 | 0.035 |
| Loc680726 | 2.64 | 4.15 | 1.57 | 0.000 |
| Map1S | 3.38 | 5.31 | 1.57 | 0.006 |
| Hip1 | 6.66 | 10.46 | 1.57 | 0.008 |
| Rin3 | 5.45 | 8.55 | 1.57 | 0.006 |
| Cadm3 | 30.17 | 47.37 | 1.57 | 0.036 |
| Dclre1B | 2.85 | 4.47 | 1.57 | 0.001 |
| Capn5 | 3.86 | 6.05 | 1.57 | 0.029 |
| Amz1 | 7.11 | 11.16 | 1.57 | 0.023 |
| Tnfrsf1B | 6.67 | 10.46 | 1.57 | 0.021 |
| Ptpn14 | 3.58 | 5.61 | 1.57 | 0.031 |
| Slc20a1 | 5.63 | 8.82 | 1.56 | 0.044 |
| Stat3 | 23.53 | 36.77 | 1.56 | 0.050 |
| Prdx4 | 27.17 | 42.46 | 1.56 | 0.041 |
| Fuca2 | 8.91 | 13.91 | 1.56 | 0.024 |
| Cytip | 5.00 | 7.81 | 1.56 | 0.024 |
| Bag3 | 8.01 | 12.47 | 1.56 | 0.024 |
| Dnase2 | 17.17 | 26.74 | 1.56 | 0.039 |
| Usp54 | 2.31 | 3.60 | 1.56 | 0.040 |
| N3 | 7.98 | 12.41 | 1.56 | 0.001 |
| C6Orf108 | 3.32 | 5.17 | 1.56 | 0.030 |
| Faim3 | 18.53 | 28.81 | 1.56 | 0.013 |
| Siva1 | 4.17 | 6.48 | 1.55 | 0.033 |
| Adamtsl4 | 10.39 | 16.15 | 1.55 | 0.021 |
| Matk | 2.35 | 3.65 | 1.55 | 0.021 |
| Fmnl1 | 6.08 | 9.44 | 1.55 | 0.010 |
| Tpm3 | 18.69 | 29.00 | 1.55 | 0.019 |
| Ywhah | 41.80 | 64.86 | 1.55 | 0.033 |
| Cerk | 11.21 | 17.38 | 1.55 | 0.042 |
| Arhgap25 | 2.14 | 3.32 | 1.55 | 0.017 |
| Galc | 2.91 | 4.51 | 1.55 | 0.035 |
| Man2B2 | 5.20 | 8.05 | 1.55 | 0.025 |
| Nipsnap3a | 7.18 | 11.11 | 1.55 | 0.017 |
| Cd55 | 23.79 | 36.82 | 1.55 | 0.024 |
| Stk10 | 6.13 | 9.48 | 1.55 | 0.020 |
| Galntl1 | 5.66 | 8.76 | 1.55 | 0.023 |
| Glrx | 14.80 | 22.87 | 1.55 | 0.032 |
| Aqp1 | 69.59 | 107.53 | 1.55 | 0.015 |
| Rab31 | 17.92 | 27.67 | 1.54 | 0.009 |
| Pold3 | 4.65 | 7.18 | 1.54 | 0.036 |
| Alcam | 8.34 | 12.86 | 1.54 | 0.044 |
| Sc65 | 3.59 | 5.53 | 1.54 | 0.036 |
| Map1B | 2.13 | 3.28 | 1.54 | 0.003 |
| Loc100364277 | 8.50 | 13.09 | 1.54 | 0.040 |
| Slc39a6 | 3.44 | 5.29 | 1.54 | 0.015 |
| Tpm3 | 47.04 | 72.36 | 1.54 | 0.023 |
| Sgms2 | 3.07 | 4.72 | 1.54 | 0.001 |
| Tug1 | 10.55 | 16.21 | 1.54 | 0.017 |
| Pcna | 25.14 | 38.62 | 1.54 | 0.005 |
| Asap1 | 8.61 | 13.22 | 1.54 | 0.014 |
| B3Gnt9 | 4.07 | 6.24 | 1.53 | 0.003 |
| Sh2B3 | 7.73 | 11.84 | 1.53 | 0.027 |
| Ptger1 | 2.89 | 4.43 | 1.53 | 0.011 |
| Znf521 | 3.95 | 6.06 | 1.53 | 0.021 |
| Cdca4 | 3.59 | 5.50 | 1.53 | 0.029 |
| Adap2 | 5.85 | 8.95 | 1.53 | 0.027 |
| Pml | 5.42 | 8.29 | 1.53 | 0.047 |
| Adamtsl5 | 2.57 | 3.94 | 1.53 | 0.023 |
| Rab3D | 9.56 | 14.62 | 1.53 | 0.033 |
| Itgav | 7.06 | 10.80 | 1.53 | 0.013 |
| Aldh1A3 | 15.54 | 23.75 | 1.53 | 0.024 |
| Lbr | 8.95 | 13.68 | 1.53 | 0.010 |
| Csf1 | 17.91 | 27.36 | 1.53 | 0.032 |
| Rabin3 | 10.49 | 16.02 | 1.53 | 0.008 |
| Spats2L | 4.17 | 6.36 | 1.53 | 0.028 |
| Sbno2 | 4.73 | 7.21 | 1.53 | 0.037 |
| Whsc1 | 4.06 | 6.19 | 1.52 | 0.002 |
| Foxp1 | 4.42 | 6.72 | 1.52 | 0.049 |
| Rps6Ka5 | 2.14 | 3.25 | 1.52 | 0.046 |
| Anxa2 | 273.32 | 415.73 | 1.52 | 0.000 |
| Prrx2 | 3.23 | 4.91 | 1.52 | 0.021 |
| Dcn | 431.34 | 655.16 | 1.52 | 0.033 |
| Banp | 4.48 | 6.80 | 1.52 | 0.023 |
| Slc25a12 | 4.63 | 7.04 | 1.52 | 0.037 |
| Lpar1 | 2.36 | 3.58 | 1.52 | 0.024 |
| Dnt1 | 2.33 | 3.53 | 1.52 | 0.044 |
| Sema4a | 4.86 | 7.36 | 1.51 | 0.041 |
| Ehd4 | 24.87 | 37.65 | 1.51 | 0.010 |
| Col5A1 | 8.67 | 13.10 | 1.51 | 0.003 |
| Rfwd3 | 2.37 | 3.57 | 1.51 | 0.001 |
| Limk1 | 4.40 | 6.64 | 1.51 | 0.017 |
| Eif2C2 | 2.50 | 3.77 | 1.51 | 0.001 |
| Tm4Sf1 | 16.21 | 24.39 | 1.50 | 0.040 |
| Tra2a | 12.98 | 19.52 | 1.50 | 0.016 |
| Hs6St1 | 13.25 | 19.92 | 1.50 | 0.014 |
| Ankrd12 | 3.43 | 5.16 | 1.50 | 0.028 |
| Acpl2 | 4.73 | 7.11 | 1.50 | 0.038 |
| Mical1 | 2.52 | 3.78 | 1.50 | 0.002 |
| Hdgfrp2 | 8.41 | 12.62 | 1.50 | 0.015 |
| *Down-regulated in LOCK* |  |  |  |  |
| Sytl5 | 5.96 | 2.14 | -2.78 | 0.027 |
| Fabp3 | 17.87 | 6.52 | -2.74 | 0.013 |
| Cish | 30.12 | 12.19 | -2.47 | 0.047 |
| Kcnip1 | 15.10 | 6.15 | -2.46 | 0.001 |
| Tmeff1 | 17.35 | 7.30 | -2.38 | 0.020 |
| Calml3 | 8.97 | 3.80 | -2.36 | 0.002 |
| Adhfe1 | 49.27 | 21.54 | -2.29 | 0.000 |
| Pmepa1 | 82.30 | 36.58 | -2.25 | 0.025 |
| Hoxa9 | 6.56 | 2.95 | -2.22 | 0.009 |
| Six1 | 8.46 | 3.93 | -2.15 | 0.008 |
| Ucp3 | 25.05 | 11.80 | -2.12 | 0.002 |
| Kifc2 | 7.37 | 3.54 | -2.08 | 0.007 |
| Cyp4F22 | 16.68 | 8.07 | -2.07 | 0.003 |
| Tbata | 6.96 | 3.41 | -2.04 | 0.015 |
| Nabp1 | 106.20 | 53.64 | -1.98 | 0.015 |
| Hoxc10 | 5.86 | 2.97 | -1.97 | 0.021 |
| Resp18 | 7.38 | 3.78 | -1.95 | 0.021 |
| Rtn4 | 270.07 | 138.89 | -1.94 | 0.002 |
| Slc25A35 | 43.72 | 22.55 | -1.94 | 0.002 |
| Camk2B | 7.13 | 3.71 | -1.92 | 0.004 |
| P2Rx5 | 24.38 | 12.75 | -1.91 | 0.001 |
| Retsat | 1387.79 | 732.96 | -1.89 | 0.009 |
| Cntf | 5.95 | 3.16 | -1.88 | 0.000 |
| Abcb9 | 6.05 | 3.28 | -1.85 | 0.027 |
| Agpat2 | 394.37 | 219.38 | -1.80 | 0.002 |
| Ccbl2 | 21.50 | 11.99 | -1.79 | 0.000 |
| Eci1 | 26.87 | 15.02 | -1.79 | 0.000 |
| Ca3 | 1326.50 | 741.59 | -1.79 | 0.001 |
| Gpx3 | 735.17 | 413.28 | -1.78 | 0.009 |
| Aig1 | 72.04 | 40.70 | -1.77 | 0.001 |
| Dbi | 5.25 | 2.97 | -1.77 | 0.002 |
| Gys2 | 30.79 | 17.56 | -1.75 | 0.038 |
| Bcat2 | 97.26 | 55.71 | -1.75 | 0.000 |
| Ppp1r1a | 61.49 | 35.25 | -1.74 | 0.000 |
| Alas1 | 52.45 | 30.17 | -1.74 | 0.016 |
| Fabp4 | 18261.06 | 10506.10 | -1.74 | 0.001 |
| Acox1 | 163.67 | 94.24 | -1.74 | 0.000 |
| Insig1 | 87.13 | 50.63 | -1.72 | 0.026 |
| Psph | 28.17 | 16.39 | -1.72 | 0.002 |
| Pla2G16 | 1129.23 | 658.95 | -1.71 | 0.001 |
| Cd300Lg | 5.12 | 2.99 | -1.71 | 0.002 |
| Ces1 | 112.58 | 66.09 | -1.70 | 0.025 |
| Gpd1 | 1861.00 | 1094.22 | -1.70 | 0.018 |
| Oxct1 | 183.75 | 108.17 | -1.70 | 0.000 |
| Rgd1309676 | 69.48 | 40.92 | -1.70 | 0.008 |
| Fdps | 23.95 | 14.11 | -1.70 | 0.028 |
| Insig1 | 611.79 | 361.49 | -1.69 | 0.025 |
| C10Orf57 | 77.45 | 45.81 | -1.69 | 0.037 |
| Cdo1 | 1446.00 | 856.06 | -1.69 | 0.000 |
| Usmg5 | 5.84 | 3.47 | -1.68 | 0.001 |
| Adig | 741.76 | 440.58 | -1.68 | 0.005 |
| Gstz1 | 61.02 | 36.29 | -1.68 | 0.003 |
| Sucnr1 | 48.80 | 29.03 | -1.68 | 0.005 |
| Rhbg | 59.92 | 35.66 | -1.68 | 0.007 |
| Ca3 | 15300.97 | 9132.66 | -1.68 | 0.001 |
| Nat8l | 54.04 | 32.26 | -1.68 | 0.001 |
| Ces1E | 23.27 | 13.89 | -1.67 | 0.042 |
| Agpat9 | 58.91 | 35.18 | -1.67 | 0.000 |
| Csad | 23.53 | 14.12 | -1.67 | 0.000 |
| Cyb5R1 | 320.07 | 192.19 | -1.67 | 0.003 |
| Rtn4 | 30.70 | 18.47 | -1.66 | 0.000 |
| Adrb3 | 93.41 | 56.27 | -1.66 | 0.005 |
| Macrod1 | 29.90 | 18.12 | -1.65 | 0.001 |
| Plekhb1 | 8.92 | 5.42 | -1.64 | 0.001 |
| Chchd10 | 307.52 | 187.66 | -1.64 | 0.017 |
| Mvd | 7.00 | 4.27 | -1.64 | 0.034 |
| Asns | 64.76 | 39.67 | -1.63 | 0.003 |
| Eci1 | 86.29 | 52.89 | -1.63 | 0.001 |
| Ces1 | 1757.97 | 1077.75 | -1.63 | 0.024 |
| Acat2 | 9.80 | 6.01 | -1.63 | 0.036 |
| Ufsp1 | 5.92 | 3.64 | -1.63 | 0.004 |
| Acot13 | 17.08 | 10.51 | -1.62 | 0.001 |
| Acat2 | 24.20 | 15.00 | -1.61 | 0.022 |
| Agt | 449.53 | 280.10 | -1.60 | 0.033 |
| Glb1L2 | 11.93 | 7.48 | -1.59 | 0.021 |
| Cidec | 979.81 | 615.20 | -1.59 | 0.000 |
| Cfd | 3993.29 | 2508.21 | -1.59 | 0.007 |
| Cmbl | 97.35 | 61.23 | -1.59 | 0.001 |
| G0S2 | 357.11 | 224.92 | -1.59 | 0.019 |
| Mpv17L | 3.45 | 2.18 | -1.59 | 0.000 |
| Hmgcs1 | 62.95 | 39.81 | -1.58 | 0.001 |
| Loc686442 | 8.07 | 5.12 | -1.58 | 0.003 |
| Rgd1566021 | 5.04 | 3.20 | -1.58 | 0.035 |
| Loc100365711 | 6.45 | 4.10 | -1.57 | 0.000 |
| Gcat | 16.01 | 10.18 | -1.57 | 0.013 |
| Pdp2 | 10.46 | 6.67 | -1.57 | 0.001 |
| Pomt2 | 24.03 | 15.36 | -1.56 | 0.001 |
| Acsl1 | 1290.07 | 825.11 | -1.56 | 0.013 |
| Nrg4 | 23.40 | 14.98 | -1.56 | 0.001 |
| Tspan12 | 210.41 | 134.85 | -1.56 | 0.017 |
| Timm10 | 17.37 | 11.18 | -1.55 | 0.012 |
| Cidea | 153.08 | 98.57 | -1.55 | 0.027 |
| Rgd1309779 | 14.54 | 9.38 | -1.55 | 0.003 |
| Hspe1 | 223.99 | 144.89 | -1.55 | 0.002 |
| Rangrf | 24.04 | 15.55 | -1.55 | 0.001 |
| Chchd3 | 6.91 | 4.48 | -1.54 | 0.000 |
| Pm20D2 | 22.10 | 14.34 | -1.54 | 0.016 |
| Atp5g3 | 91.71 | 59.59 | -1.54 | 0.002 |
| Cd36 | 634.18 | 412.68 | -1.54 | 0.000 |
| Chchd2 | 657.04 | 428.46 | -1.53 | 0.001 |
| Kcnh2 | 18.34 | 11.97 | -1.53 | 0.002 |
| Desi1 | 4.73 | 3.09 | -1.53 | 0.027 |
| Adipor2 | 139.32 | 91.11 | -1.53 | 0.022 |
| Csad | 57.75 | 37.77 | -1.53 | 0.001 |
| Rnf125 | 21.73 | 14.21 | -1.53 | 0.012 |
| Loc100996724 | 28.73 | 18.84 | -1.53 | 0.018 |
| Acox1 | 88.49 | 58.13 | -1.52 | 0.016 |
| Mtch2 | 15.08 | 9.92 | -1.52 | 0.007 |
| Acp5 | 41.91 | 27.56 | -1.52 | 0.011 |
| Loc152217 | 52.49 | 34.54 | -1.52 | 0.002 |
| Rbm47 | 12.88 | 8.48 | -1.52 | 0.024 |
| Eif4g3 | 3.57 | 2.36 | -1.51 | 0.048 |
| Immp2l | 4.94 | 3.27 | -1.51 | 0.033 |
| Hsp40 | 66.95 | 44.38 | -1.51 | 0.000 |
| Phyh | 409.64 | 271.63 | -1.51 | 0.000 |
| Qdpr | 152.63 | 101.22 | -1.51 | 0.001 |
| Dbo | 143.23 | 95.13 | -1.51 | 0.016 |
| S100A1 | 17.35 | 11.53 | -1.50 | 0.005 |
| Cyc1 | 16.43 | 10.93 | -1.50 | 0.010 |
| Cdkn2c | 60.00 | 39.92 | -1.50 | 0.007 |
| Pxmp2 | 4.38 | 2.91 | -1.50 | 0.021 |
| Mrps10 | 20.14 | 13.42 | -1.50 | 0.006 |
| Fitm2 | 57.30 | 38.18 | -1.50 | 0.002 |
