# Supplementary material for: Rapid Alterations in Perirenal Adipose Tissue Transcriptomic Networks with Cessation of Voluntary Running
Source: PLoS One. 2015 Dec 17;10(12):e0145229. doi: 10.1371/journal.pone.0145229 (PMC4683046; doi:10.1371/journal.pone.0145229)
Supplement: S2 Table — (DOCX) [file pone.0145229.s002.docx]

S2 Table: Complete list of transcripts expressed only in RUN, sorted by magnitude of RPKM.

| **Transcript Description** | **RUN RPKM** |
| --- | --- |
| Rfx4 | 2.44 |
| Hey2 | 2.25 |
| Gipr | 2.09 |
| Tshr | 2.01 |
| Grtp1 | 1.86 |
| Nckap5 | 1.75 |
| Ace2 | 1.67 |
| Clmp | 1.60 |
| Pde4C | 1.52 |
| Neu2 | 1.48 |
| Bzrap1 | 1.46 |
| Thra | 1.30 |
| Dda1 | 1.29 |
| Ces1 | 1.29 |
| Bend7 | 1.28 |
| Leo1 | 1.27 |
| Grtp1 | 1.26 |
| Psmb3 | 1.25 |
| Slc1A1 | 1.25 |
| Aar2 | 1.25 |
| Cdc42 | 1.21 |
| Clmp | 1.17 |
| Ppm1B | 1.17 |
| Itgae | 1.14 |
| Mbp | 1.13 |
| Pcdh17 | 1.12 |
| Tmem79 | 1.11 |
| Hddc3 | 1.10 |
| Six4 | 1.09 |
| Mon1A | 1.07 |
| Ostn | 1.04 |
| Plekhb1 | 1.03 |
