# Supplementary material for: Rapid Alterations in Perirenal Adipose Tissue Transcriptomic Networks with Cessation of Voluntary Running
Source: PLoS One. 2015 Dec 17;10(12):e0145229. doi: 10.1371/journal.pone.0145229 (PMC4683046; doi:10.1371/journal.pone.0145229)
Supplement: S3 Table — (DOCX) [file pone.0145229.s003.docx]

S3 Table: Complete list of transcripts expressed only in LOCK, sorted by magnitude of RPKM.

| **Transcript Description** | **LOCK RPKM** |
| --- | --- |
| Top2A | 7.42 |
| Cdkn3 | 7.13 |
| Mki67 | 5.87 |
| Tpx2 | 5.11 |
| Nusap1 | 5.01 |
| Ccna2 | 4.85 |
| Racgap1 | 4.58 |
| Ckap2 | 4.50 |
| Aurkb | 4.32 |
| Kif23 | 4.16 |
| Birc5 | 3.92 |
| Rprm | 3.89 |
| Cdca3 | 3.83 |
| C15Orf23 | 3.76 |
| Cdc20 | 3.72 |
| Mcm5 | 3.65 |
| Ndc80 | 3.32 |
| Pttg1 | 3.26 |
| Nuf2 | 3.05 |
| Ptx3 | 2.87 |
| Kif11 | 2.86 |
| Ncaph | 2.78 |
| Dctd | 2.77 |
| Spc25 | 2.60 |
| Fam64A | 2.56 |
| Vcan | 2.54 |
| Tgif2 | 2.51 |
| Osr2 | 2.50 |
| Cep55 | 2.47 |
| Gpsm2 | 2.45 |
| Pmaip1 | 2.44 |
| Pbk | 2.41 |
| Cenpa | 2.40 |
| Asf1B | 2.39 |
| Cdca7 | 2.32 |
| Aldh1L2 | 2.31 |
| Atp8B4 | 2.30 |
| Loc503326 | 2.28 |
| Sox12 | 2.27 |
| Gins1 | 2.24 |
| Clec4D | 2.24 |
| Foxm1 | 2.23 |
| Plk1 | 2.21 |
| Esco2 | 2.16 |
| Bub1B | 2.16 |
| Ncapg | 2.11 |
| Ddx11 | 2.08 |
| Fcn1 | 2.07 |
| Rnase6 | 2.06 |
| Iqgap3 | 2.05 |
| Dbf4 | 2.02 |
| Fanci | 1.99 |
| Plk1 | 1.98 |
| Tmem107 | 1.98 |
| Insc | 1.93 |
| Dfna5 | 1.93 |
| Rad51Ap1 | 1.90 |
| Cdt1 | 1.90 |
| Cyp7B1 | 1.90 |
| Adam8 | 1.90 |
| Pkmyt1 | 1.88 |
| Cenpo | 1.88 |
| Loc728392 | 1.86 |
| Ntsr1 | 1.86 |
| Dmkn | 1.86 |
| Ccdc111 | 1.86 |
| Rad51 | 1.84 |
| Mctp1 | 1.80 |
| Ttk | 1.78 |
| Psrc1 | 1.76 |
| Dsn1 | 1.76 |
| Wdhd1 | 1.75 |
| Fam54A | 1.74 |
| Ckap2L | 1.72 |
| Fancd2 | 1.72 |
| Itgax | 1.72 |
| Zpld1 | 1.70 |
| Cdc25C | 1.69 |
| Cenpm | 1.68 |
| Ticam2 | 1.67 |
| Mxd3 | 1.66 |
| Cenpw | 1.65 |
| Sgk3 | 1.65 |
| Mn1 | 1.65 |
| Diaph3 | 1.64 |
| Cenpe | 1.63 |
| Ankrd33B | 1.63 |
| Kif15 | 1.63 |
| Nek2 | 1.62 |
| Snap25 | 1.62 |
| Batf | 1.61 |
| Cx3Cr1 | 1.61 |
| Depdc7 | 1.60 |
| Sema3E | 1.60 |
| Slc1A4 | 1.60 |
| Mex3B | 1.59 |
| Veph1 | 1.58 |
| Melk | 1.56 |
| Clcf1 | 1.56 |
| Atp1A3 | 1.56 |
| Prkcb | 1.55 |
| Opn3 | 1.55 |
| Casc5 | 1.55 |
| Spsb4 | 1.55 |
| Ccdc46 | 1.53 |
| Efna2 | 1.52 |
| Chek1 | 1.52 |
| Kif18B | 1.52 |
| Aspm | 1.51 |
| Nme4 | 1.50 |
| Prr11 | 1.50 |
| Troap | 1.50 |
| Slc25A13 | 1.49 |
| Slc6A9 | 1.49 |
| Kcnt2 | 1.47 |
| Fjx1 | 1.47 |
| Scamp5 | 1.47 |
| Rad51C | 1.45 |
| Cass4 | 1.44 |
| Rhamm | 1.44 |
| Rgd1561161 | 1.43 |
| Bmp2K | 1.43 |
| Ccdc99 | 1.42 |
| Znf862 | 1.42 |
| F2Rl1 | 1.42 |
| Wnt9A | 1.42 |
| Treml2 | 1.41 |
| Cdc45 | 1.41 |
| Stx11 | 1.40 |
| Ccnf | 1.40 |
| Ttll13 | 1.40 |
| Camk2G | 1.38 |
| Efr3B | 1.37 |
| Hells | 1.36 |
| Eppk1 | 1.36 |
| Sgol2 | 1.35 |
| Hdac9 | 1.35 |
| Uhrf1 | 1.35 |
| Chtf18 | 1.35 |
| Nphp3 | 1.33 |
| Ska1 | 1.33 |
| Tmem150B | 1.32 |
| Bard1 | 1.32 |
| Mthfd1L | 1.31 |
| Ccsap | 1.31 |
| Mlf1Ip | 1.30 |
| Loc100363169 | 1.30 |
| Agphd1 | 1.29 |
| Slit2 | 1.28 |
| Mastl | 1.28 |
| Wdfy4 | 1.28 |
| Kif20B | 1.28 |
| Hivep3 | 1.27 |
| Piezo2 | 1.26 |
| Shcbp1 | 1.25 |
| Trip13 | 1.25 |
| Kiaa0825 | 1.25 |
| Ncf2 | 1.23 |
| Mms22L | 1.23 |
| Card14 | 1.23 |
| Fhl1 | 1.23 |
| Defa1B | 1.23 |
| Loc100365744 | 1.23 |
| Kif2C | 1.22 |
| Vwf | 1.22 |
| Map7D2 | 1.22 |
| Chml | 1.22 |
| Osbpl10 | 1.22 |
| Rgd1562052 | 1.22 |
| Kiaa1755 | 1.22 |
| Ccr3 | 1.21 |
| Prkacb | 1.21 |
| Mns1 | 1.21 |
| Gdf15 | 1.21 |
| Phka2 | 1.20 |
| Ube2T | 1.20 |
| Chaf1B | 1.20 |
| Fam70A | 1.20 |
| Gas2L3 | 1.20 |
| Spata2L | 1.19 |
| Rab15 | 1.19 |
| Prdm9 | 1.19 |
| Tnfsf15 | 1.18 |
| Cntrob | 1.18 |
| Dgkh | 1.18 |
| Siglec-3 | 1.18 |
| Nav2 | 1.17 |
| Ccdc19 | 1.17 |
| Slc1A7 | 1.17 |
| Loc682147 | 1.17 |
| Inpp4B | 1.17 |
| C1Orf112 | 1.17 |
| Ttc8 | 1.17 |
| Psg7 | 1.16 |
| Pole | 1.16 |
| Clec4A | 1.16 |
| Pld1 | 1.15 |
| Dclk2 | 1.15 |
| Kcnd1 | 1.14 |
| Kif18A | 1.14 |
| Rbpjl | 1.14 |
| Mypop | 1.14 |
| Rgd1563245 | 1.14 |
| Homez | 1.13 |
| Ero1Lb | 1.13 |
| Bub3 | 1.13 |
| Loc691236 | 1.12 |
| Mcm8 | 1.11 |
| Phf19 | 1.11 |
| Sncaip | 1.11 |
| Gef | 1.11 |
| Ephb3 | 1.10 |
| Cdca2 | 1.10 |
| Adrbk2 | 1.10 |
| Brsk1 | 1.10 |
| Depdc1 | 1.10 |
| Naip | 1.09 |
| Cenpq | 1.09 |
| Znf287 | 1.08 |
| Arhgap33 | 1.08 |
| Gjc2 | 1.08 |
| Fam122B | 1.07 |
| Mmp25 | 1.06 |
| Loc691241 | 1.06 |
| Znf384 | 1.05 |
| Gins3 | 1.05 |
| Fancc | 1.05 |
| Ston2 | 1.04 |
| C14Orf79 | 1.04 |
| Arsj | 1.04 |
| Dok2 | 1.04 |
| Loc687065 | 1.03 |
| Cenpi | 1.03 |
| Adcy10 | 1.03 |
| Qpct | 1.02 |
| Cntn2 | 1.02 |
| Fzd5 | 1.02 |
| Brunol5 | 1.02 |
| Fam83D | 1.02 |
| Plxdc1 | 1.01 |
| Chst3 | 1.01 |
| Ccnb2 | 1.01 |
| Rae-1Gamma | 1.00 |
| Ptprc | 1.00 |
